# Factors related to acupuncture response in patients with chronic severe functional constipation: Secondary analysis of a randomized controlled trial

**DOI:** 10.1371/journal.pone.0187723

**Published:** 2017-11-22

**Authors:** Xingyue Yang, Yan Liu, Bing Liu, Liyun He, Zhishun Liu, Yanshi Yan, Jia Liu, Baoyan Liu

**Affiliations:** 1 Beijing University of Chinese Medicine, Beijing, China; 2 Institute of Basic Research in Clinical Medicine, China Academy of Chinese Medical Sciences, Beijing, China; 3 Institute of Acupuncture and Moxibustion, China Academy of Chinese Medical Sciences, Beijing, China; 4 Guang’an Men Hospital, China Academy of Chinese Medical Sciences, Beijing, China; University Hospital Llandough, UNITED KINGDOM

## Abstract

**Background:**

Acupuncture has been demonstrated to be effective and safe for chronic severe functional constipation (CSFC). However, which patients with CSFC will have a better response to acupuncture remains unclear.

**Objective:**

To explore factors related to acupuncture response in patients with CSFC.

**Methods:**

We performed a secondary analysis of a previous multicenter randomized controlled trial consisting of a 2-week run-in period, 8-week treatment, and 12-week follow-up without treatment in which patients with CSFC were randomly allocated to an electroacupuncture group or a sham electroacupuncture group. Responders were defined as participants with an increase of at least one complete spontaneous bowel movement (CSBM) in week 20 compared with the baseline period. The CSBM responder rate in both groups was described, and the baseline characteristics of participants potentially related to acupuncture response were mainly analyzed using logistic regression analysis with bootstrapping techniques.

**Results:**

A total of 1021 participants were analyzed in this study, of whom 516 (50.5%) were classified as responders. The CSBM responder rate in week 20 was significantly greater in the electroacupuncture group than in the sham electroacupuncture group (62.9% vs. 37.9%, respectively; P<0.001). Both age and comorbidity were negatively associated with clinical response: with every one-year increase in age, the likelihood of clinical response was reduced by 1.2% (OR 0.988, 95%CI 0.980 to 0.996; P = 0.005), and patients with comorbidities were approximately 42% less likely to respond to treatment (OR 0.581, 95%CI 0.248 to 0.914; P = 0.001).

**Conclusion:**

CSFC patients with increasing age and comorbidity may be less likely to respond to acupuncture. These findings contribute to guiding clinical practice in terms of pretreatment patient selection. Further research is needed to confirm the association.

## Introduction

Chronic constipation is a common symptom that can influence the patient’s quality of life. Patients associate constipation with several symptoms: infrequent bowel movements, hard or lumpy stools, frequent straining, bloating, a feeling of incomplete evacuation after a bowel movement, and abdominal discomfort [1]. Chronic constipation affects nearly 16% of the world population [2] and is higher in women, nonwhites, individuals aged >65 years and individuals with lower socioeconomic status [3]. Constipation is usually caused by many factors such as diet, medications, metabolic or neurological disorders, and psychosocial issues, as well as dysfunction of colonic motility and the defecation process [3].

Acupuncture has been accepted as an effective treatment for functional gastrointestinal diseases in the West [4]. However, the therapeutic effects of acupuncture remain controversial. Some trials have demonstrated that acupuncture was no more effective than sham acupuncture [5, 6]. One of the most important reasons is that the therapeutic effects of acupuncture are multifactorial, including both specific and non-specific factors [7]. Acupuncture is a complex intervention, and some people are more likely to respond than others [8, 9]. However, there are few studies exploring which patients are more likely to benefit from acupuncture, while most clinical studies focus on the evaluation of acupuncture effectiveness. The question of which factors influence patient response to acupuncture needs to be addressed.

In our previous research [10], our results indicated that an eight-week period of electroacupuncture was effective and safe for chronic severe functional constipation (CSFC). In this study, we performed a post hoc secondary analysis to explore factors that might influence the acupuncture response in patients with CSFC.

## Methods

### Overview of the original trial

The detailed protocol has been described in our published article [11] (See S1 Text). Therefore, in this article, we briefly summarize the protocol below. The study was a multicenter, randomized, parallel, sham-controlled trial at 15 hospitals in China between 8 October 2012 and 4 May 2014, consisting of a 2-week run-in period, an 8-week treatment with electroacupuncture or sham electroacupuncture, and a 12-week follow-up without treatment. A total of 1075 patients were enrolled (last recruited on 4 May 2014): 536 in the electroacupuncture group and 539 in the sham electroacupuncture group. A total of 54 patients dropped out during the study, and 1021 patients remained in week 20. The primary outcome was the change from the baseline in mean weekly complete spontaneous bowel movements (CSBMs) during weeks 1 to 8. The secondary outcomes were the changes from the baseline in mean CSBMs per week during weeks 9 to 20, the mean spontaneous bowel movements per week during weeks 1 to 8, the mean Bristol stool form scale score during weeks 1 to 8, the mean patient assessment of constipation quality of life (PAC-QOL) score at weeks 4 and 8, the proportion of participants with ≥3 CSBMs per week and the proportion of participants using rescue measures for constipation. Adverse events during the trial were also observed.

This study protocol was approved by the Ethics Committee (See S2 Text), and the trial was conducted in compliance with the Declaration of Helsinki and the Good Clinical Practice guidelines. Every participant was well informed and signed the informed consent form. The trial was registered at ClinicalTrials.gov (NCT01726504).

### Secondary analysis design

The data on both the electroacupuncture and the sham electroacupuncture group were analyzed in this study. We compared the CSBM responder rate in the two groups. The CSBM responder rate was defined as the proportion of participants with an increase of at least one CSBM from the baseline [12]. The baseline value was defined as the average number of CSBMs per week during the 2-week screening period (weeks -1 and -2). Responders and non-responders were classified based on the increase in the number of CSBMs in week 20 compared with the baseline value.

We considered the possibility that the baseline characteristics of the participants might account for the acupuncture response. These factors consisted of baseline demographic characteristics, including treatment assignment, age, gender, race, body mass index (BMI), and some indicators associated with CSFC, including constipation duration, CSBMs per week, PAC-QOL score and comorbidity. The baseline mean CSBMs per week indicated the patients’ bowel function. The mean PAC-QOL score indicated the effects of constipation on physical discomfort, psychosocial discomfort, worriedness and concern, and satisfaction in their daily lives.

### Statistical analysis

The demographic and clinical characteristics of patients, including treatment assignment, age, gender, race, BMI, constipation duration, comorbidity, CSBMs per week, and mean PAC-QOL score, were summarized by descriptive statistics. We conducted a baseline univariate analysis of the data on the aforementioned variables between the responder group and the non-responder group. For continuous variables, comparisons between groups were assessed using Student’s *t* test. Categorical variables were compared using the chi-square test, and the Wilcoxon rank-sum test was used to analyze ordinal variables. The variables that were significant at the 0.25 level [13] in the univariate analyses were selected as candidates for a multivariable logistic regression model. We assessed the interactions between the treatment assignment and the candidate variables introduced into the multivariable logistic regression model. Backward elimination model-building with a bootstrap approach (using 1000 bootstrap samples of all the data) was used to select independent variables to be retained in the final model [14].

Before the regression analyses, the presence of collinearity among the predictor variables was checked using the variance inflation factor (VIF). We considered collinearity to be present if the VIFs of the variables were >5. All statistical analyses were performed using SAS version 9.4 (SAS Institute) and R version R3.4.1 (R Foundation for Statistical Computing, Vienna, Austria) with a 2-sided P value of less than 0.05 considered significant.

## Results

### CSBM responder rate from week 1 to week 20

**Fig 1:**
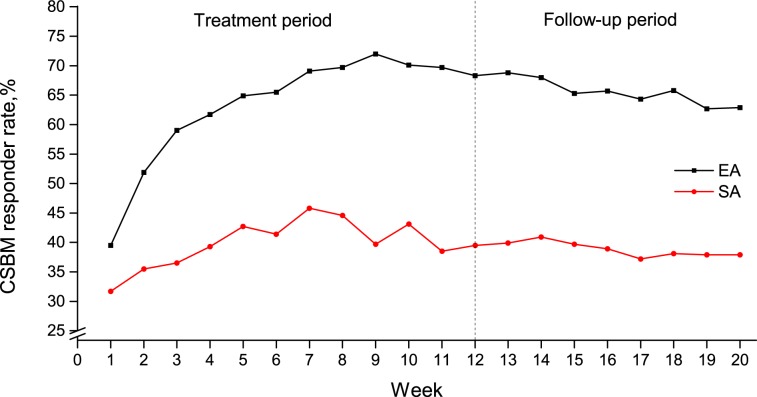
CSBM responder rate in EA and SA groups from week 1 to week 20. The differences in the CSBM responder rate between the two groups were significant in both the treatment and follow-up periods (*P*<0.01). CSBM = complete spontaneous bowel movement; EA = electroacupuncture; SA = sham electroacupuncture.

A total of 1021 participants were included in the full analysis set, of whom 516 (50.5%) were classified as responders. The baseline characteristics of the whole population were described in our original research [8]. Fig 1 shows the CSBM responder rate from week 1 to 20. Compared with the sham acupuncture group, the electroacupuncture group had a higher CSBM responder rate. From week 2 onward, the CSBM responder rate of the electroacupuncture group was more than twice that of the sham electroacupuncture group. It is worth mentioning that in week 9, the CSBM responder rate in the electroacupuncture group was 72.0%, compared with 39.7% in the sham electroacupuncture group (See S1 Table). Participants in the electroacupuncture group had a better sustained response to acupuncture than that of the sham electroacupuncture group after the treatment period.

### Logistic regression analysis of related factors in acupuncture responders

The baseline characteristics of the responders are shown in Table 1, including their demographic and clinical characteristics. Logistic regression analysis with backward elimination identified 3 of 15 factors (8 candidate variables and 7 interactions between the candidate variables and treatment assignment) as significantly associated with acupuncture responders: treatment assignment, age and comorbidity (Table 2). The odds of CSBM responders in the electroacupuncture group were 2.82 times higher than in the sham electroacupuncture group (OR 0.355, 95%CI 0.094 to 0.615; *P*<0.001). Increased age was associated with non-responders, as the likelihood of response decreased by 1.2% with each one-year increase in age (OR 0.988, 95%CI 0.980 to 0.996; *P* = 0.005). More importantly, comorbidity was significantly associated with acupuncture non-responders even after controlling for age. Patients with comorbidities were approximately 42% less likely to be CSBM response than patients with no comorbidity (OR 0.581, 95%CI 0.248 to 0.914; *P* = 0.001). It was evident that participants without comorbidity showed better improvement than patients with any other diseases in the CSBM response, and younger patients tended to show better improvement than the aged. In addition, the interaction terms between treatment assignment and the key baseline factors were not statistically significant, and did not make it into the final model. No collinearities were observed among variables in the multivariable logistic regression analysis (VIFs <1.5). After bootstrapping and adjustment for overfitting, the ROC area of the final model was 0.652 (overfitting 0.0349). Table 2 (columns 2 and 5) shows the regression coefficients and odds ratio per predictor adjusted for overfitting (i.e., after bootstrapping).

**Table 1:** Demographic and clinical characteristics of responders ^a^.

Characteristics	Responders (N = 516)	Non-responders (N = 505)	Total (N = 1021)	*P* Value ^b^
Treatment assignment				<0.001
Electroacupuncture	324 (62.8)	191 (37.8)	515 (50.4)	
Sham electroacupuncture	192 (37.2)	314 (62.2)	506 (49.6)	
Age, mean (SD), y	44.8 (16.10)	49.0 (15.92)	46.9 (16.14)	<0.001
Gender				0.082
Male	110 (21.3)	131 (25.9)	241 (23.6)	
Female	406 (78.7)	374 (74.1)	780 (76.4)	
Race				0.140
Han	498 (96.5)	495 (98.0)	993 (97.3)	
Non-Han	18 (3.5)	10 (2.0)	28 (2.7)	
BMI (kg/m^2^)				0.156
≤18.5	40 (7.8)	25 (5.0)	65 (6.4)	
>18.5 to ≤23.9	324 (62.8)	333 (65.9)	657 (64.3)	
>23.9 to ≤27.9	135 (26.2)	123 (24.4)	258 (25.3)	
>27.9	17 (3.3)	24 (4.8)	41 (4.0)	
Constipation duration, y				0.009
≤10	362 (70.2)	298 (59.0)	660 (64.6)	
>10 to ≤20	93 (18.0)	124 (24.6)	217 (21.3)	
>20 to ≤30	40 (7.8)	56 (11.1)	96 (9.4)	
>30 to ≤40	14 (2.7)	20 (4.0)	34 (3.3)	
>40 to ≤50	4 (0.8)	5 (1.0)	9 (0.9)	
>50	3 (0.6)	2 (0.4)	5 (0.5)	
CSBMs				0.101
≤1	469 (90.9)	443 (87.7)	912 (89.3)	
>1 to ≤3	47 (9.1)	62 (12.3)	109 (10.7)	
PAC-QOL score				0.543
≤2	92 (17.8)	77 (15.2)	169 (16.6)	
>2 to ≤3	253 (49.0)	269 (53.3)	522 (51.1)	
>3 to ≤4	149 (28.9)	139 (27.5)	288 (28.2)	
>4 to ≤5	22 (4.3)	20 (4.0)	42 (4.1)	
Comorbidity				<0.001
Yes	85 (16.5)	140 (27.7)	225 (22.0)	
No	431 (83.5)	365 (72.3)	796 (78.0)	

Abbreviations: BMI, body mass index; CSBM, complete spontaneous bowel movement; PAC-QOL, patient assessment of constipation quality of life.

^a^ Data are expressed as no. of participants (%) unless otherwise indicated.

^b^ A threshold of P<0.25 was used to select variables [13].

**Table 2:** Backward logistic regression with bootstrap method.

Variables	B	SE	*P* Value	Odds Ratio (95% CI)^a^
Intercept	1.233	0.222	<0.001	
Treatment assignment	-1.037	0.133	<0.001	0.355 (0.094 to 0.615)
Age	-0.012	0.004	0.005	0.988 (0.980 to 0.996)
Comorbidity	-0.543	0.170	0.001	0.581 (0.248 to 0.914)

^a^ Regression coefficient and corresponding odds ratio after bootstrapping (i.e., adjusted for overfitting).

## Discussion

This secondary analysis of this randomized controlled trial showed that age and comorbidity were the potential factors related to acupuncture response for CSFC patients. Younger patients and patients without coexisting diseases had higher CSBM responder rates. Factors such as gender, race, BMI, constipation duration, CSBM per week, and PAC-QOL score had no relevant influence on acupuncture response. (See S2 Table)

According to previous research, an increase in CSBM per week ≥1 is considered to be a clinical response [12]. The electroacupuncture and sham electroacupuncture groups had different proportions of responders in week 20, namely, 62.9% and 37.9%, respectively. Previous trials reported that the mean proportion of CSBM responders upon treatment with 2 mg and 4 mg of prucalopride once a day was 47.3% and 46.6%, respectively, compared with 25.8% in the placebo group [15]. Participants in the sham electroacupuncture group received shallow needling at non-acupoints with mock electrostimulation, which is a commonly used method to ensure the blinding of participants [16]. The results were consistent with previous findings that this sham electroacupuncture was inferior to real acupuncture for patients with gastrointestinal disorders [4, 17]. However, other research has reported no difference between sham electroacupuncture and electroacupuncture for some pain diseases [18]. Some non-specific factors may be possible explanations for acupuncture response, or the difference might be related to the diseases.

Previous studies have shown that many factors can influence acupuncture response, such as baseline demographic characteristics, disease severity, acupuncture stimulation, patient expectations and the interaction between the patient and the acupuncturist [19, 20].

Regarding the increases in constipation, its prevalence is associated with gender, race, age, and socioeconomic status, among other factors. Patients aged above 65 have a higher prevalence of constipation, reaching up to 70% [21]. Studies have demonstrated that colonic motility changes in both human and animals with increasing age [22, 23]. Bhavik Anil Patel *et al*. showed that fecal output and water content were reduced in aged animals and that age increased the availability of mucosal 5-hydroxytryptamine and tumor necrosis factor expression and decreased mucosal serotonin transporter expression and the availability of 5-hydroxyindoleacetic acid [24]. Moreover, constipation in elderly patients is also related to other factors, such as pelvic floor aging, decreased social activity, psychological disorders, comorbidity, and the effects of multiple drug usage [25]. Therefore, patients with increasing age have a worse response to acupuncture. The treatment of age-related chronic constipation is challenging and requires more clinical research to provide effective treatment approaches.

In gender, race, and BMI, there were no significant differences between responders and non-responders. Previous research on prucalopride for chronic constipation patients also indicated that there was no significant difference between men and women in efficacy outcomes [26], which was similar to our results. As this trial was conducted in China, race comparison was performed mainly between Han and other minorities who were all of Chinese ethnicity. There is to date no research showing differences between Han Chinese and minorities.

Factors related to the severity of chronic constipation, such as constipation duration, CSBMs per week, and PAC-QOL score, also showed no difference between responders and non-responders. There was previous research showing that patients with more severe baseline dysfunction were likely to benefit from acupuncture [27]. Although there was in this study no statistical significance for these three factors in relation to the severity of chronic constipation, Table 2 shows more responders with constipation duration ≤10 years than non-responders, namely, 70.2% vs 59.0%, while for patients with constipation duration >10 years, there are fewer responders than non-responders. As this work is a secondary analysis of a randomized controlled trial, the participants included in this trial all had severe constipation, defined as CSBMs<3 per week, which did not address all levels of mild, moderate and severe constipation. This participant selection might explain this result and will need further exploration.

Patients without comorbidity were more likely than patients with comorbidity to be responders. One possible explanation for our findings was that the inner *qi* of patients with multiple diseases was out of condition because of dysfunction in the visceral sensation or mobility [28]. From the perspective of traditional Chinese medicine, the main function of acupuncture was to activate the inner *qi* and regulate the bowel function [29]. Therefore, individuals with comorbidities had worse responses to acupuncture. From another perspective, constipation is associated with a large number of diseases and is an adverse effect of many drugs [1]. Comorbidities or drugs taken were, to a certain extent, likely aggravating chronic constipation.

This RCT includes a large sample in the acupuncture field, which is significant for deeper analysis using this mass of data. Acupuncture response in patients is still an under-studied field and needs further exploration to identify individuals who might benefit from acupuncture. This preliminary finding could guide clinical practice in pretreatment patient selection.

However, this secondary analysis has several limitations. Since we used only the factors collected in the original protocol, some potential factors that might relate to acupuncture response, such as psychological factors, acupoints, and stimulation parameters, could not be explored. Other potential demographic factors, such as education level, occupation, economic status, and history of gastrointestinal disease were also not involved. Second, our study was conducted only in China, and the conclusions might be different in other counties or worldwide, especially regarding some social-demographic factors. Third, as the study was a secondary analysis of the RCT, some results were difficult to interpret, as it was not clear whether the analyses were statistically underpowered for finding a small association or whether no association existed.

## Conclusion

Patients show a better response to electroacupuncture than sham electroacupuncture, and this improved response lasts for 12 weeks after the treatment period. CSFC patients with increasing age and coexisting illness may be less likely to respond to acupuncture. However, this conclusion still requires further confirmation.

## Supporting information

S1 Text Detailed protocol of original research. (PDF)

S2 Text Ethics committee approval files. (PDF)

S1 Table CSBM responder rate from week 1 to week 20. (DOC)

S2 Table CONSORT checklist. (DOC)
