# Supplementary material for: Factors related to acupuncture response in patients with chronic severe functional constipation: Secondary analysis of a randomized controlled trial
Source: PLoS One. 2017 Nov 22;12(11):e0187723. doi: 10.1371/journal.pone.0187723 (PMC5699843; doi:10.1371/journal.pone.0187723)
Supplement: S1 Text — (PDF) [file pone.0187723.s001.pdf]

**The efficacy and safety of electro-acupuncture for severe chronic functional constipation: original study protocol for a multicenter, randomized, sham-controlled trial**

**Clinical Sites:**

1. Guang'anmen Hospital Affiliated to China Academy of Chinese Medical Sciences
2. West China Hospital of Sichuan University
3. Yueyang Hospital of Integrated Traditional Chinese and Western Medicine, Shanghai University of Traditional Chinese Medicine
4. The Third Affiliated Hospital of Zhejiang Chinese Medical University
5. Guangdong Province Hospital of TCM
6. The First Affiliated Hospital of Tianjin University of Traditional Chinese Medicine
7. Jiangsu Province Hospital of TCM
8. Beijing Traditional Chinese Medicine Hospital Affiliated to Capital Medical University
9. Heilongjiang Province Academy of Chinese Medical Science
10. The First Affiliated Hospital of Anhui University of Chinese Medicine
11. Wuhan Integrated TCM & Western Medical Hospital
12. Nanjing University of Chinese Medicine
13. Dongzhimen Hospital Affiliated to Beijing University of Traditional Chinese Medicine
14. Huguosi Hospital of Chinese Medicine Affiliated to Beijing University of Chinese Medical Science
15. 301 Hospital

**Data Management and Statistical Centers:**

1. Institute of Basic Research in Clinical Medicine, China Academy of Chinese Medical Sciences
2. Biostatistics Center of the University of Washington (Seattle)

**Downloaded From: http://annals.org/ by a China Academy of Chinese Medical Sciences User on 09/13/2016**

## Table of Contents

**1. Study Contact and Organization** .......... 3
**1.1 Study Contacts** .......... 3
**1.2 Recruiting Sites** .......... 3
**1.3 Collaborating Sites** .......... 5

**2. Study Design** .......... 5
**2.1 Study Overview** .......... 5
**2.2 Background** .......... 5
**2.3 Study Objectives and Hypothesis** .......... 6
**2.4 Methodology** .......... 6

**3. Safety Assessment** .......... 11
**3.1 Adverse Events** .......... 11
**3.2 Report of the Severe Adverse Event (SAE)** .......... 12
**3.3 Safety related to EA** .......... 12

**4. Interventions** .......... 12
**4.1 Electro-acupuncture (EA)** .......... 12
**4.2 Sham electro-acupuncture (SA)** .......... 13
**4.3 Rescue Medicine** .......... 14

**5. Informed consent** .......... 14

**6. Quality Control** .......... 17
**6.1 Quality Control** .......... 17
**6.2 Quality Guarantee** .......... 17
**6.3 Compliance Improvement** .......... 18
**6.4 Data and Safety Monitoring Board (DSMB)** .......... 18

**7. Data Management** .......... 18
**7.1 Flow Chart of the Data Management** .......... 18
**7.2 The Raw Data Management and Archiving** .......... 19
**7.3 Data Entry and Storage** .......... 19
**7.4 Data Verification and Problems Solving** .......... 19
**7.5 Medical Coding** .......... 19
**7.6 Data Report** .......... 20
**7.7 Data Auditing and Blinding Review** .......... 20
**7.8 Database Locking** .......... 20

**8. Data Analysis** .......... 20
**8.1 Objectives and Hypothesis** .......... 20
**8.2 Statistical Analysis Population** .......... 20
**8.3 Statistical Contents and Methods** .......... 20

**9 Ethical principle** .......... 21

**10 Funding** .......... 21

**11 References** .......... 21

**12 Updates on the original protocol** .......... 22

**Appendix—DSMB for Data and Safety Monitoring Plan (DSMP)** .......... 23



Downloaded From: http://annals.org/ by a China Academy of Chinese Medical Sciences User on 09/13/2016

**1. Study Contact and Organization**

**1.1 Study Contacts**


**<u>Institute of Basic Research in Clinical Medicine, China Academy of Chinese Medical Sciences</u>**

Baoyan Liu, MD (**Principal Investigator for CHOICE grant)**
16 Nanxiaojie Dongzhimennei, Dongcheng, Beijing, China, 100700
Phone: +86 13601180524
Email: 13601180524@139.com

Liyun He, MD PhD
16 Nanxiaojie Dongzhimennei, Dongcheng, Beijing, China, 100700
Phone: +8610 64014411-2408
Email: heliyun@tcmcec.com

**<u>Guang'anmen Hospital of China Academy of Chinese Medical Sciences</u>**

Zhishun Liu, MD PhD (**Principal Investigator for trial**)
Director of the Acupuncture Department
5 Beixian'ge Street, Xicheng District, Beijing, China, 100053
Phone: +86 10 88001123
Email: liushizhun@aliyun.com

Yuying Cai, MD PhD
Acupuncture Department
5 Beixian'ge Street, Xicheng District, Beijing, China, 100053
Phone: +8610 88001033
Email: bjcaiyuying@163.com

Jiani Wu, MD
Acupuncture Department
5 Beixian'ge Street, Xicheng District, Beijing, China, 100053
Phone: +86 10 88001123
Email: handsom_mars@126.com


**1.2 Recruiting Sites**


**<u>Guang'anmen Hospital of China Academy of Chinese Medical Sciences:</u>**

Huanfang Xu, MD PhD
Acupuncture Department
5 Beixian'ge Street, Xicheng District, Beijing, China, 100053
Phone: +8610 88001123
Email: 15711082019@126.com

Ruosang Du, MD PhD
Acupuncture Department
5 Beixian'ge Street, Xicheng District, Beijing, China, 100053
Phone: +86 10 88001123
Email: 605119619@qq.com

**<u>West China Hospital of Sichuan University</u>**

Ning Li, MD
Acupuncture Department
37 Guoxuexiang, Wuhou District, Chengdu, Sichuan, 610041
Email: zhenjiuhuaxi@163.com

**<u>Yueyang Hospital of Integrated Traditional Chinese and Western Medicine, Shanghai University of Traditional Chinese Medicine</u>**

Guirong Dong, MD
Acupuncture Department




Downloaded From: http://annals.org/ by a China Academy of Chinese Medical Sciences User on 09/13/2016

110 Ganhe Road, Shanghai, China, 200437
Email: dongguirong2000@126.com

**The Third Affiliated Hospital of Zhejiang Chinese Medical University**
Jianqiao Fang, MD PhD
Acupuncture Department
219 Moganshan Road, Hangzhou, China, 310005
Phone: +86 0571 86673000
Email: fangjianqiao7532@163.com

**Guangdong Province Hospital of TCM**
Wenbin Fu, MD
Acupuncture Department
111 Dade Road, Yuexiu District, Guangzhou, Guangdong, China, 510120
Phone: +86 020 81887233-34230
Email: fuwenbin@139.com

**The First Affiliated Hospital of Tianjin University of Traditional Chinese Medicine**
Lixin Fu, MD PhD
Acupuncture Department
314 Anshan W Road, Nankai District, Tianjin, China, 300193
Phone: +86 022 27432095
Email: fulixin66@126.com

**Jiangsu Province Hospital of TCM**
Jianhua Sun, MD PhD
Acupuncture Department
155 Hanzhong Road, Qinhuai District, Nanjing, Zhejiang, China, 210029
Phone: +86 025 86617141
Email: drjhsun@sina.com

**Beijing Traditional Chinese Medicine Hospital Affiliated to Capital Medical University**
Linpeng Wang, MD
Acupuncture Department
23 Meishuguanhou Street, Dongcheng District, Beijing, China, 100010
Phone: +86 13911406703
Email: wlp5558@sina.com

**Heilongjiang Province Academy of Chinese Medical Science**
Shun Wang, MD
33 Xidazhi Street, Nangang District, Ha'erbin, Heilongjiang, 150080
Phone: +86 13115318866
Email: hljwang@aliyun.com

**The First Affiliated Hospital of Anhui University of Chinese Medicine**
Jun Yang, MD
Acupuncture Department
117 Meishan Road, Hefei, Anhui, China, 230031
Phone: +86 055 12838581
Email: yangjunacup@126.com

**Wuhan Integrated TCM & Western Medical Hospital**
Hongxing Zhang, MD
Acupuncture Department
215 Zhongshan Avenue, Qiaokou District, Wuhan, Hubei, China, 430022
Phone: +86 027 82770520



Downloaded From: http://annals.org/ by a China Academy of Chinese Medical Sciences User on 09/13/2016

Email: zhxzj99@aliyun.com

**Nanjing Traditional Chinese Medicine Hospital Affiliated to Nanjing University of Chinese Medicine**
Jianbin Zhang, MD PhD
282 Hanzhong Road, Gulou District, Nanjing, Zhejiang, China, 210029
Phone: +86 025 85811658
Email: zhangjianbin@njutcm.edu.cn

**Dongzhimen Hospital Affiliated to Beijing University of Traditional Chinese Medicine**
Jiping Zhao, MD
Acupuncture Department
5 Haiyuncang, Dongcheng District, Beijing, China, 100700
Phone: +86 10 84013147
Email: ZJP7883@sina.com

**Huguosi Hospital of Chinese Medicine Affiliated to Beijing University of Chinese Medical Science**
Wei Zhou, MD
Acupuncture Department
83 Mianhua Hutong, Xicheng District, Beijing, China, 100035
Phone: +86 10 83224261-2155
Email: dzhouwei@sina.com

**301 Hospital**
Ling Guan, MD PhD
Acupuncture Department
28 Fuxing Road, Haidian District, Beijing, China, 100853
Phone: +86 13520575830
Email: guanling301@sina.com

**1.3 Collaborating Sites**

**Institute of Basic Research in Clinical Medicine, China Academy of Chinese Medical Sciences**
Shiyan Yan, PhD
16 Nanxiaojie Dongzhimennei Dongcheng, Beijing, China, 100700
Phone: +86 13521436209
Email: yanshiyan0927@sina.com

Yanke Ai, PhD
16 Nanxiaojie Dongzhimennei Dongcheng, Beijing, China, 100700
Phone: +86 13521601603
Email: aiyanke@163.com

**Daemen College Physical Therapy Wound Care Clinic, Daemen College**
Kehua Zhou, MD DPT
4380 Main Street, Amherst, N.Y. 14226, USA
Phone: + 1 716-986-4856
Email:kzhou@daemen.edu

**2. Study Design**

**2.1 Study Overview**

The objective of study is to assess the efficacy and safety of electro-acupuncture (EA) for symptoms associated with severe functional constipation.

**2.2 Background**

Constipation is a common gastrointestinal disease. The prevalence of chronic functional constipation is 14.7% in the USA[1] and 11.6% in Asia.[2] Patients with severe chronic constipation have complete spontaneous bowel movements (CSBMs) no more than twice per week, with hard stools, and the frequency of staining is usually more than 1/4.[3] The disorder is so intractable that nearly half of patients are



Downloaded From: http://annals.org/ by a China Academy of Chinese Medical Sciences User on 09/13/2016

dissatisfied with their laxative therapies.[4] Despite an instant effect, long-term use of medicine may lead to some side effects such as drug dependence, melanosis coli, laxative-induced constipation, or even cancerization[3]. Systematic reviews indicate that acupuncture might be effective for constipation; however, the evidence is limited.[5] Our pilot studies showed deep needling electro-acupuncture (EA) worked from week 1, which was quicker than either shallow needling (worked from week 2) or medication with lactulose (Duphalac). The treatment group adopted deep needling at Tianshu (ST25), who showed a frequency of weekly spontaneous defecation of 2.75±1.48 (M±SD), compared with the shallow needling group (0.79±0.93) and the medication group (2.17±1.15) after 4 weeks of treatment. This study indicated that deep needling EA at ST25 might be superior to shallow puncture and Duphalac, but further research is needed to provide more evidencet.[6,7] In addition, these studies had significant limitations which include lack of information about the severity of patients' constipation symptoms at baseline and the assessing endpoints limited to the last week of treatment or follow-up. By comparison, in this trial, patients with severe constipation will be treated for 8 weeks, and followed up for 12 weeks by assessing the mean weekly value of outcomes during the 8-week treatment and 12-week follow-up. In addition, this trial is a multicenter randomized controlled trial, and aims to evaluate the safety and effectiveness of EA for severe constipation.

**2.3 Study Objectives and Hypothesis**

Study objective is to assess the efficacy and safety of EA, and to test the hypothesis that the efficacy of EA is better than that of sham electro-acupuncture (SA) in patients with chronic severe functional constipation. In addition, the sustained effect of EA will also be evaluated.

Hypothesis:

The group receiving EA treatment will have a statistically significant and clinically important improvement over the group receiving SA.

**2.4 Methodology**

**2.4.1 Trial Design**

This is a 15-site, two-arm, randomized controlled trial, which will be performed from Oct 1, 2012 to Nov 30, 2014 (Figure 1). All the acupuncturists in each site are required to have an official license and clinical work experience no less than 2 years. In this trial, the participants, evaluators, and statisticians will be blinded.

**2.4.2 Subjects**

Participants must meet the diagnostic criteria for functional constipation according to Rome III[8]: (1) must include ≥2 of the following: 1) straining during ≥25% of defecations, 2) lumpy or hard stools in ≥25% of defecations, 3) sensation of incomplete evacuation for ≥25% of defecations, 4) sensation of anorectal obstruction /blockage for ≥25% of defecations, 5) manual maneuvers to facilitate for ≥25% of defecations, 6) <3 defecations per week; (2) loose stools rarely present without use of laxatives; (3) insufficient evidence for irritable bowel syndrome. To meet the criteria of functional constipation, ≥2 of these symptoms must have been present for ≥ 3 months, with symptoms onset ≥ 6 months before diagnosis.

**2.4.2.1 Inclusion Criteria**

Participants will be included if they fulfill all of the followings: (1) Meeting the diagnosis of Rome III criteria[8] for chronic functional constipation; (2) Severe chronic constipation with two or fewer complete spontaneous bowel movements (CSBMs) per week for more than 3 months; (3) Aged 18 to 75 years old; (4) No use of medicine for constipation 2 weeks before enrollment (except rescue medication), no acupuncture treatment for constipation in the previous 3 months, and had not joined any other trial in the previous 3 months; (5) Volunteered to join this research and signed the informed consent.

**2.4.2.2 Exclusive Criteria**

Participants will be excluded if they met any of the followings: (1) constipation caused by irritable bowel syndrome (IBS), or drugs, or secondary to endocrine, metabolic, nervous or postoperative diseases; (2) Constipation accompanied with serious cardiovascular, hepatic, or renal damage, or with cognitive dysfunction, psychiatric disorder, aphasia, or severe dystrophy, which might affecting the cooperation for examination or treatment; (3) women in gestation or lactation periods; (4) Constipation accompanied with abdominal aneurysm, hepatosplenomegaly; (5) Bleeding disorders, or regular anticoagulant drug users, such as warfarin, heparin, etc.; (6) Cardiac pacemaker carrier.

**2.4.2.3 Subject Withdrawals**

The participants may leave the study at their own discretion, or the experts from the gastrointestinal or anorectal department in each site may determine that it is the best for the participants to discontinue participation (due to worsening of symptoms, or the occurrence of a serious adverse event). Members of



Downloaded From: http://annals.org/ by a China Academy of Chinese Medical Sciences User on 09/13/2016

our data and safety monitoring board (DSMB) of the trial will take charge of the data monitoring and safety, and will decide if the whole clinical trial should be aborted according to the evaluation of severe adverse effects.

**2.4.2.4 Subject Recruitment, Screen and Grouping**

Participants with severe functional constipation will be recruited through posters, or advertisements on newspapers, websites, or on TV. Research assistants of each site will preliminarily screen the participants by recording their disease condition, history of the disease and treatment, and the demographic data. Physicians from the gastrointestinal or anorectal department of each site will take charge of the diagnosis and the differential diagnosis of the severe functional constipation. Potential participants will fill out a 2-week screening defecation diary to record their bowel movements, constipation-related symptoms, medications, etc. Eligible participants then will be randomized to EA or SA group. Acupuncturists are in charge of the participants' assignment, and the EA or SA manipulation. They are also responsible for the assessment of safety during treatment. Research assistants take care of the diet instruction for the participants, and let them keep the diet and daily routines as usual. They also require the participants to stop their medicine for constipation. During the trial, the professional evaluators of each site will instruct the participants how to fill in their defecation diaries and patients' self-assessment related to the trial and the evaluators will record the data on the case report form (CRF) through the whole trial period.

**2.4.3 Flow Chart**



Downloaded From: http://annals.org/ by a China Academy of Chinese Medical Sciences User on 09/13/2016

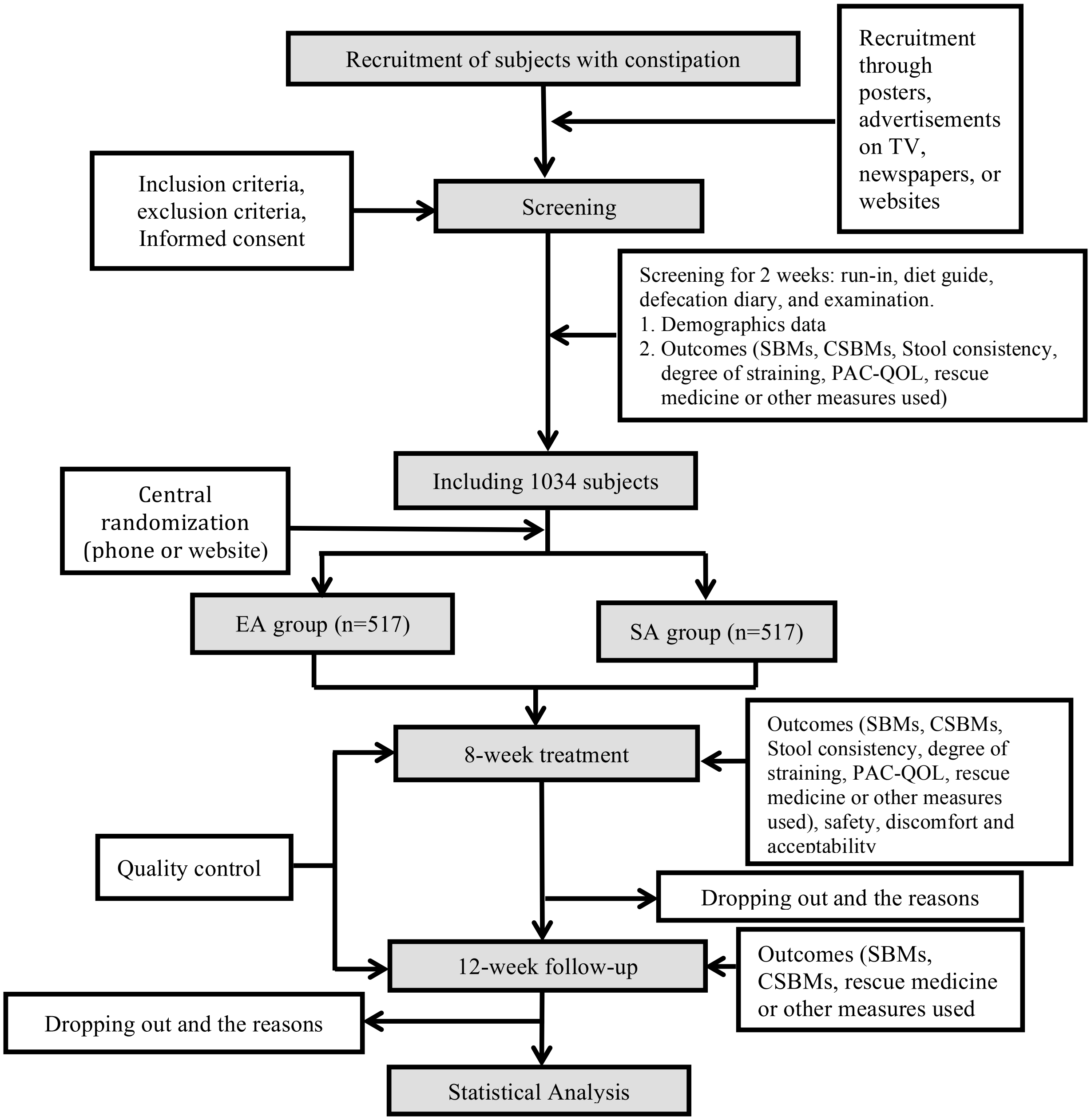


**Figure 1 Flow Chart**

**2.4.4 Sample Size**

According to our pilot study, the weekly CSBMs increase was conservatively estimated at 1.49 times, with a standard deviation of 1.77, after 8 weeks EA treatment; the weekly CSBMs increased by 1.01, with a standard deviation of 1.61, after 8 weeks SA treatment. We used SAS 9.3 to define the 95% power, using a one-way analysis of variance fixed effects omnibus test, assuming a 20% loss to follow-up. To ensure the stratified randomization, the sample size calculation result is 1034.

1034 participants were enough to provide 95% power to detect a difference of 0.5 in the change of weekly CSBMs between the two groups at a significance level of 5% using analysis of variance. The intergroup difference of 0.5 weekly CSBMs was within the range of published estimates of the minimum clinically important difference (MCID) for weekly CSBMs.[9,10]

**2.4.5 Randomization**

Participants will be randomized to EA and SA groups with a ratio of 1:1 by using the central stratified block randomization. The site will be set as the stratified factor. The professional biostatisticians will



Downloaded From: http://annals.org/ by a China Academy of Chinese Medical Sciences User on 09/13/2016

generate the randomization schedule and related document by using SAS 9.3 software, PROC PLAN. A central randomization system (Institute of Basic Research in Clinical Medicine, China Academy of Chinese Medical Sciences) will be adopted. The researchers who take charge of the randomization will input the birthdate and gender of the eligible subjects through telephone or website to get the random numbers. Experts who will generate the randomization schedule from the Institute of Basic Research in Clinical Medicine, China Academy of Chinese Medical Sciences will take no park in the statistical analysis. The randomization schedule and the related parameters will be sealed and signed by the experts and keep by the staff that doesn't participate in our research. No one has the right to check the randomization schedule.

**2.4.6 Blinding**

The participants, evaluators, and statisticians will be blinded. Participants do not know which intervention they will be given. The evaluators from each site who is blinded about the assignment will take charge of the outcome assessment. And the statistician who is blinded about assignment from Institute of Basic Research in Clinical Medicine, China Academy of Chinese Medical Sciences will be responsible for the statistical analysis.

**2.4.7 Outcomes Measurement**

**2.4.7.1 Primary Outcome**

The change of the mean weekly complete spontaneous bowel movements (CSBMs)[3] from baseline over the 8-week treatment:

$$\frac{\text{the total number of CSBMs over the 8 weeks of treatment}}{8} - \frac{\text{the total number of CSBMs over the 2 weeks of baseline}}{2}$$

Assessing points: baseline, at the end of week 8.

The professional evaluators totalize the CSBMs according to patients' defecation diaries.

**2.4.7.2 Secondary Outcomes**

(1) The proportion of patients having ≥ 3 CSBMs/week over 8-week treatment, assessing points: baseline, at the end of week 8. The CSBMs/week of the patients with severe constipation are ≤ 2 at baseline; therefore, the proportion of patients having ≥ 3 CSBMs/week can reveal the clinical effectiveness.

(2) The proportion of patients having ≥ 3 CSBMs/week over 12-week follow-up, assessing points: baseline, at the end of week 20.

(3) The change of the mean weekly CSBMs from baseline over the 12-week follow-up:

$$\frac{\text{the total number of CSBMs over the 12 weeks of follow}-\text{up}}{12} - \frac{\text{the total number of CSBMs over the 2 weeks of baseline}}{2}$$

Assessing points: baseline, at the end of week 20.

(4) The change of the mean weekly spontaneous bowel movements (SBMs) from baseline over the 8-week treatment:

$$\frac{\text{the total number of SBMs over the 8 weeks of treatment}}{8} - \frac{\text{the total number of SBMs over the 2 weeks of baseline}}{2}$$

Assessing points: baseline, at the end of week 8.

The professional evaluators totalize the SBMs according to patients' defecation diaries.

(5) The change of the mean score of stool consistency during each SBM from baseline over the 8-week treatment:

$$\frac{\text{sum of the score of stool consistency over the 8 weeks of treatment}}{\text{frequency of SBMs over the 8 weeks of treatment}} - \frac{\text{sum of the score of stool consistency over the 2 weeks of baseline}}{\text{frequency of SBMs over the 2 weeks of baseline}}$$



Downloaded From: http://annals.org/ by a China Academy of Chinese Medical Sciences User on 09/13/2016

Assessing points: baseline, at the end of week 8.
Participnats will report the stool consistency during their each SBM according to the 7-type BSFS[11] (scored by 1 to 7 respectively).

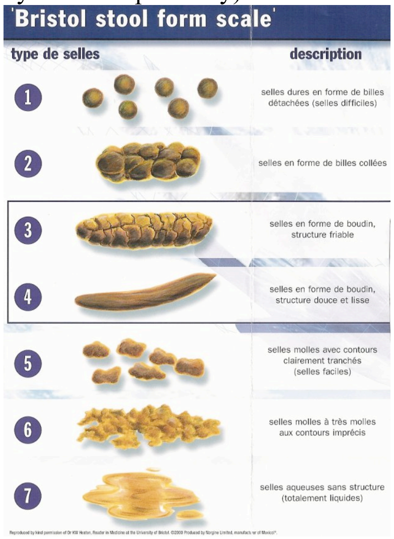


- Type 1: Separate hard lumps, like nuts (hard to pass)
- Type 2: Sausage-shaped, but lumpy
- Type 3: Like a sausage but with cracks on its surface
- Type 4: Like a sausage or snake, smooth and soft
- Type 5: Soft blobs with clear cut edges (passed easily)
- Type 6: Fluffy pieces with ragged edges, a mushy stool
- Type 7: Watery, no solid pieces. Entirely liquid

6) The change of the mean score of straining during each SBM from baseline over the 8-week treatment:

$$\frac{\text{sum of the score of straining of every SBM over the 8 weeks of treatment}}{\text{frequency of SBMs over the 8 weeks of treatment}} - \frac{\text{sum of the score of straining of every SBM over the 2 weeks of baseline}}{\text{frequency of SBMs over the 2 weeks of baseline}}$$

Assessing points: baseline, at the end of week 8.
Participants will report the degree of straining during their each SBM in the defecation diaries.

- 0 = not difficult
- 1 = a little difficult, need some straining to defecate
- 2 = difficult, need straining to defecate
- 3 = very difficult, need hard straining to defecate

(7) The change of the score of PAC-QOL from baseline at week 8.
Patient Assessment of Constipation Quality of Life questionnaire (PAC-QOL)[12] is a self-report questionnaire for evaluating the quality of life in participants with constipation, which was distributed by Mapi Research Trust in France. This questionnaire contains 28 items including 4 basic parts of physical discomfort, worries and concerns, psychosocial discomfort, and satisfaction. We will use the Chinese version in our trial.[13]
Assessing points: baseline, at the end of week 4 and week 8.
(8) The proportion of rescue medicine (glycerol enema, or sorbitol enema) used over the 8-week treatment and 12-week follow-up, and the mean weekly frequency of rescue medicine used during the same time frame; the proportion of other assistant methods used for constipation during the same time frame, and the mean weekly frequency of other assistant methods used during the same time frame.
Assessing point: baseline, at week 4, week 8, and week 20.
(9) Subgroup analysis
The subgroup analysis on the primary outcome by stratified the participants as < 65 years and ≥65 years will be conducted.
Assessing points: baseline, at the end of week 8.

**Outcomes Measurement**

Outcomes include the complete spontaneous bowel movements (CSBMs), spontaneous bowel movements (SBMs), stool consistency (Bristol Stool Scale), straining during the defecation, rescue medicine or other measures used, and the questionnaire of Patient Assessment of Constipation Quality Of Life (PAC-QOL). Patients will record their bowel movements, constipation-related symptoms, medicine used for constipation, and daily diet 24 hours per day for 22 weeks.



Downloaded From: http://annals.org/ by a China Academy of Chinese Medical Sciences User on 09/13/2016

The SBMs are the bowel movements that occur in the absence of rescue medicine, or other assistant methods (laxatives, enemas, suppositories usage, or digging out by fingers) within the preceding 24 hours, which include the CSBMs. When participants used rescue medicine, or other measures for constipation, the defecation within 24 hours was considered a non-spontaneous bowel movement, whereas defecation exceeding 24 hours was deemed a SBM. CSBMs were the SBMs with the sensation of complete evacuation.
Outcome record time point: Baseline (week 0), week 2, week 4, week 6, week 8, week 12, week 16, and week 20.
Outcome assessing time point: week 4, week 8, and week 20.
Outcomes assessing is based on the patients' defecation diaries from the first week of screening period (week -2) until the last week of the follow-up period (week 20).

**2.4.7.3 Defecation Diaries:**

Defecation diaries during the treatment period (week 1–week 8):
Participants will record their bowel movements (BMs), SBMs, complete defecating or incomplete defecating, stool consistency, degree of straining, the time, dosage, and frequency of rescue medicine or other assistant methods used, and the abnormal diet or not on the diaries every day from the baseline to the end of week 8. Distribution of the defecation diaries: the diary of week 1 will be distributed at the first treatment of week 1, and the diary of week 2 will be distributed at the last treatment of week 1; the diary of week 1 will be taken back at the first treatment of week 2, and the diary of week 3 will be distributed at the last treatment of week 2; and so on. The diary of weeks 9–12 (follow-up period) will be distributed at the last treatment of week 8. Participants should return the diary of week 8 within week 9.
Defecation diaries during the follow-up period (week 9–week 20):
Participants will record their bowel movements (BMs), SBMs, complete defecating or incomplete defecating, and the time, dosage, frequency of rescue medicine or other assistant methods used on the diaries every day from the first of week 9 to the end of week 20. Distribution of the defecation diaries: research assistants should distribute 4-week diaries at once. The diaries of weeks 9–12 will be distributed at the last treatment of week 8. Within week 9, participants should return the diary of week 8, and get the diaries of weeks 13–16; within week 13, participants should return the diaries of weeks 9–12, and get the diaries of weeks 17–20; within week 17, participants should return the diaries of weeks 13–16; within one week after week 20, participants should return the diaries of weeks 17–20.
For the participants who do not abide by our treatment protocol strictly, the outcome evaluators should still follow them up through phone, text or a visit to record the related items.

**2.4.7.4 The success of participants blinding**

We will evaluate the rate of the successful blinding between EA and SA in 140 subjects (70 from EA group, and 70 from SA group; 9 to 10 participants at each site), who will be selected at random from our participants. After five minutes of the subjects' last treatment of week 4 and week 8, they will be asked if they have received the EA treatment. We will compare the proportions of the patients choosing EA treatment between the two groups.

**3. Safety Assessment**

**3.1 Adverse Events**

The adverse events are some unintended symptoms, physical signs, or health conditions that show up after starting the interventions. A health condition or an illness existing before the treatment can be reported as an adverse event (AE) if the situation becomes worse after the treatment. An abnormal result of the laboratory examination can be reported as an AE only if it causes some clinical symptoms, and requires related therapies. We will observe the adverse events by asking the participants' non-inducing questions at each assessing point. We will also observe the adverse events through participants' self-report, or through physical examination, or by laboratory examination. All the adverse events must be recorded on the CRF. If an AE was observed, the following information should be provided:

1. Degree of severity (mild, moderate, severe);
2. If it is related to the interventions (affirmably, very likely, possibly, possibly not, affirmably not);
3. Duration of the AE (start and end dates, whether still exists at the end of the trial);
4. Severe adverse effect (SAE) or not.

SAE is defined as any of the following:

1. Fatal or life threatening;
2. Permanent or conspicuous loss of function or disability;
3. Congenital deformity or birth defect;
4. Need hospitalization or need prolonged hospitalization;



Downloaded From: http://annals.org/ by a China Academy of Chinese Medical Sciences User on 09/13/2016

5. Severe medical accidents, which require medical or surgical interventions to prevent any of the above occurs.

Solution for controlling the adverse events:

- Further observing, without any treatment;
- Adjusting or stopping the treatment;
- Aborting the trial permanently;
- Adding combined drugs therapy;
- Giving non-pharmacologic therapy;
- Hospitalizing or prolonging hospitalization.

All the treatment measures for the adverse events should be recorded in detail on the CRF. We will follow up the adverse event through the whole trial until it is solved. We will confirm that if it is permanent. At every assessing point, we will evaluate the adverse event's severity, relation to our interventions, and the treatment measures. The common adverse events related to the interventions are listed in the investigator's brochure. During the updating of the brochure, related adverse events will be announced by the form of investigator's note (IN). We will also discuss with the participants about the adverse events that might occur during the trial.

**3.2 Report of the Severe Adverse Event (SAE)**

For participants' safety, from the day they sign the informed consent until within 30 days after the end of the study, any SAE no matter if it is related to our interventions should be reported to Huanfang Xu (Project management office, Acupuncture Department, Guang'anmen Hospital of China Academy of Chinese Medical Sciences; +86 15711082019) within 24 hours. SAE occurs over 30 days after the end of the study will not require reporting, unless it is related to our interventions. The reoccurrence, complication, or the progression of a reported SAE will be reported as the follow-up information of that SAE within 24 hours. All the information of the SAE will be collected and recorded on the form of SAE. Researchers should assess the relation of the SAE with our interventions, and fax the finished SAE form to the department of Integrated Medical Safety (IMS) of our project at Guang'anmen Hospital within 24 hours. The SAE form and the fax will be kept together with the CRF in the research center.

Safety Evaluation:

1. Safe, no adverse event, no abnormal examination of the safety index;
2. Relatively safe, mild adverse event with no treatment needed, no abnormal examination of the safety index;
3. Having safety problems, moderate adverse event which needs relative treatment, mildly abnormal examination of the safety index;
4. Withdrawal from the study due to the severe adverse event, or obviously abnormal examination of the safety index.

**3.3 Safety related to EA**

Adverse events will be recorded and measured both by participants themselves and acupuncturists. Acupuncturists are responsible for the recording of the EA-related adverse events on the CRF. The adverse events relating to acupuncture include broken needle, needle left in the body after treatment, nausea during acupuncture, fainting caused by needling during the treatment, pricking lasted more than half an hour (no matter how much the intensity is) after acupuncture, hematoma or bleeding around the site of needling, numbness or infection around the site of needling, sleeplessness after acupuncture, dizziness after acupuncture, other discomforts (including palpitation, headache, asitia, drowsiness, or aggravation of existing symptoms) after acupuncture. Acupuncturists will record the symptoms, frequency, degree, and the duration of each AE. The pricking caused by needling will be assessed through 10-point visual analog scale (VAS). We will compare the proportion of participants who get acupuncture-related adverse effects, and the frequency of occurrence of each adverse effect between EA and SA groups. Participants who get acupuncture-related adverse effects at least once will be counted in for the comparison of the proportion.

**4. Interventions**

Needle instrument: Huatuo disposable needles (size 0.30mm×25mm, 0.30 mm×40mm, 0.30 mm×50mm, 0.35mm×75mm, Suzhou Medical Appliance, Suzhou, Jiangsu Province, China),

Electro-acupuncture apparatus: Huatuo electro-acupuncture apparatus (type SDZ-V, Suzhou Medical Appliance, Suzhou, Jiangsu Province, China) will be used.

**4.1 Electro-acupuncture (EA)**

Acupoints:



Downloaded From: http://annals.org/ by a China Academy of Chinese Medical Sciences User on 09/13/2016

Bilateral Tianshu (ST 25), Fujie (SP 14), Shangjuxu (ST 37).
Location:
According to the WHO Standardized Acupuncture Points Location[14].
Manipulation:
After sterilizing the skin in participants with supine position, needles of the size of 0.30 mm×50 mm, or 0.35 mm×75 mm will be inserted into ST25 and SP14 slowly and vertically, without manipulation, for approximately 30 to 70 mm until they pierced the muscle layer of the abdominal wall. Paired alligator clips of the EA apparatus will be attached transversely to the needle holders of the bilateral ST25 and SP14. EA stimulation will last for 30 minutes with a dilatational wave of 10/50Hz and current intensity of 0.1 to 1 mA. The skin around the acupoints shivering mildly indicates the proper dose. Needles of the size of 0·30 mm×40 mm will be inserted in to ST37 vertically for about 25 to 30 mm. Three small equal manipulations of twirling, lifting and thrusting will be performed. The participants' feeling of sore and distention show the proper manipulation. The manipulation on ST37 mentioned above should be performed every 10 minutes, three times in 30 minutes.
Course of treatment:
Each participant will accept an 8-continuous-week treatment with 28 sessions in total. The participants will be treated 30 minutes once, once a day, five times per week in the first two weeks, and three times per week in the latter 6 weeks.

**4.2 Sham electro-acupuncture (SA)**

Acupoints:
Bilateral sham-Tianshu (sham-ST25), sham-Fujie (sham-SP14), and sham-Shangjuxu (sham-ST37)
Location:
Sham-ST25 locates laterally and horizontally from ST25, in the middle of the spleen and stomach channels. Sham-SP14 lies laterally and horizontally from ST14, in the middle of the spleen and stomach channels. Sham-ST37 stands laterally and horizontally from ST37, in the middle of the stomach and gallbladder channels.
Manipulation:
After skin disinfection in participants with supine position, needles of the size of 0.30 mm×25 mm will penetrate the skin uprightly at approximately 3–5 mm without any manipulation. Paired alligator clips of the specially made EA apparatus (power output lines, which connect the alligator clips and the EA apparatus, will be cut inside with an appearance as usual) will be attached transversely to the needle holders of the bilateral sham-ST25 and sham-SP14. The EA apparatus will be turned on with a working power indicator and the same sound as the EA group. These will suggest the participants that the EA apparatus is working, however there is no current output. Needles of the size of 0.30 mm×25 mm will penetrate into bilateral sham-ST37 vertically at about 3–5 mm without any manipulation.
Course of Treatment:
The course is the same as EA.
All the participants will be treated separately in order to prevent their communication with each other. Our 22-week trial contains 2 weeks of screening period, 8 weeks of treatment period, and 12 weeks of follow-up period (Figure 2). Collecting points of the outcomes: week -2 (screening), week 0 (baseline), week 2 (treatment), week 4 (treatment), week 6 (treatment), week 8 (treatment), week 12 (follow-up), week 16 (follow-up), week 20 (follow-up).



Downloaded From: http://annals.org/ by a China Academy of Chinese Medical Sciences User on 09/13/2016

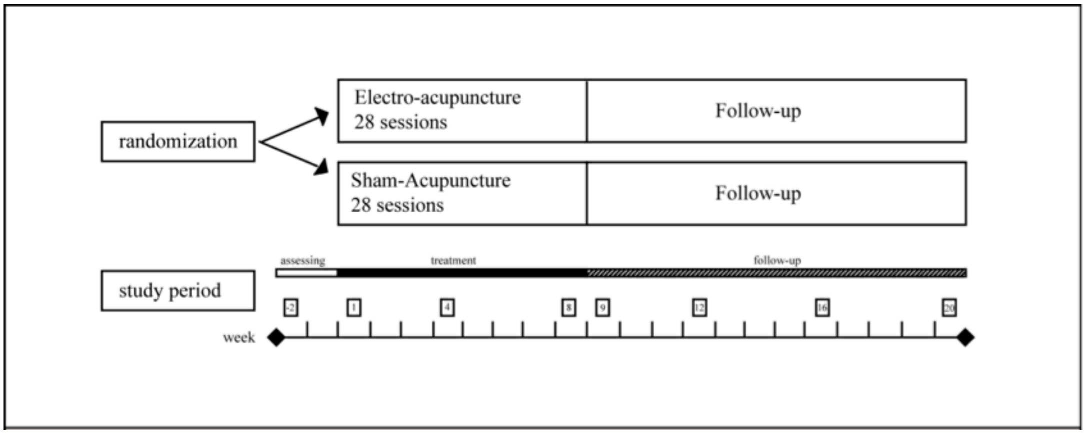


**Figure 2 The study period and assessing timeframe of our trial**

**4.3 Rescue Medicine**

If the participants don't have a bowel movement for 3 or more consecutive days during the whole trial period, they are allowed to use glycerol enema 110ml, or sorbitol enema 40–60ml once into anus as a rescue medicine. The time, frequency, and dosage of the rescue medicine used should be recorded at length on participants' defecating diaries. Other drugs for constipation are not allowed in our trial. However, if the participants have used any other drug, they must faithfully record on their diaries the name, time, frequency, and dosage of the drug used.

**5. Informed consent**

Informed Consent: Study Introduction

Dear participants：

If your doctor thinks you have severe functional constipation, we invite you to participate in the study on the evaluation of effectiveness and safety of acupuncture for treating severe functional constipation. This study is supported and funded by the "the 12th Five-Year Program'' of the National Science and Technology Pillar Program (2012BAI24B01). The purpose of this study is to evaluate the effectiveness and safety of acupuncture for treating severe functional constipation.

Before your decision to participate in the study, please read the following information carefully. It is helpful for you to know this study, understand why the study is performed, the study procedures, the duration and benefits of the study, risks and potential discomforts during and after study participation. If you prefer, you can also discuss this study with your relatives and friends, or consult medical doctors whom your trust for explanation and help to make the decision.

Introduction

I. Background and purposes

The prevalence of constipation is high, and it affects around 11.6%–14.7% of the general population. Although western medicine usually introduces immediate relief for constipation, its long-term use can lead to side effects including drug dependence, melanosis coli, constipation due to laxative abuse, or even cancer. Currently, there are no known satisfactory treatments available, especially for severe chronic constipation. A systematic review shows that acupuncture may be effective for treating chronic constipation and our previous pilot study indicates that electro-acupuncture may be effective for treating severe chronic constipation with long-lasting effects and a good safety profile. In this study, a randomized controlled trial design was used and we aim to evaluate the effectiveness, advantages of efficacy, and safety of acupuncture treatment for severe functional constipation. This study will be carried out simultaneously in fifteen class A tertiary hospitals all over China, and we expect a total number of 1080 participants for voluntary participation.

II. Exclusion criteria

(1) Secondary constipation caused by other diseases;
(2) Younger than 18 or older than 75;
(3) Patients who are confused and cannot express his/her subjective symptoms, and patients with psychiatric diseases;



Downloaded From: http://annals.org/ by a China Academy of Chinese Medical Sciences User on 09/13/2016

(4) Patients with progressive malignant tumors or other serious wasting diseases and prone to infection and hemorrhage;
(5) Patients with co-morbidities of other serious primary diseases including cardiovascular, hepatic, renal, digestive, hematopoietic system diseases;
(6) Pregnant and lactating women.

III. What to do next, if you decide to participate?
1. Before your enrollment in the study, you will receive the following exams to determine whether you are eligible to participate in the study:
The doctor will ask and record your medical history and perform related physical examination.
You will be required to get lower gastrointestinal tract radiography or colonoscopy, stool routine test, and others for the confirmation of your diagnosis.
2. If the results of the above screening examinations meet the inclusion criteria and you are willing to participate in this study, you will be invited to continue study participation in the following steps:
(1) Based on the random number generated from the computer, the doctor will assign you to either the electro-acupuncture (EA) or sham electro-acupuncture (SA) groups.
(2) In the study, Huatuo brand disposable needles (Suzhou Medical Appliance, Jiangsu, China, Jiangsu Food, Drug, and Medical Appliance Administration production approval No.: 2001–0020, Registration No: 2270202 in Year 2004) will be used. Needle size: 0·30×25mm, 0·30×40mm, 0·30×50mm, indicating the diameter of needle is 0.30mm and the length of needle is 1 Cun, 2 Cun, and 3 Cun, respectively. Huatuo's EA apparatus will be used.
(3) The duration of this study is 22 weeks, including a baseline period of 2 weeks, a treatment period of 8 weeks, and a follow-up period of 12 weeks. Frequency and duration of EA treatment: five sessions in each of the first 2 weeks, followed by 3 sessions per week in week 3 to 8, once every 2 days. The patients will receive 28 sessions of treatment in total.
(4) During the study period, you need to record detailed symptom diary faithfully (time, frequency, and degree of difficulty of daily bowel movements, characteristics of the stool, time and dosage of drug use if any, etc.). After treatment, you will need to hand in your diary to the doctor timely, and the doctor will record your signs and symptoms in detail.
3. Other requirements for your cooperation
As a participant of this study, you will have some relevant responsibilities, such as adherence to the schedule for examination, treatment, and outpatient follow-up. Additionally, you are also responsible for reporting any changes in your physical and mental status to your doctor during the study process regardless of whether you think these changes are related to the study or not.
You should follow the scheduled appointments with the doctor to come to the hospital for treatment (during follow-up, the doctor may get to know your conditions by phone or visiting your home). Your follow-up is very important because the doctor will determine whether the treatment that you are receiving really works, and the doctor will be able to guide the prevention and management of your symptoms timely.
During the study, you are not allowed to use other medications for constipation. However, if you do not have a bowel movement for three or more consecutive days during the study, you are permitted to take Glycerol Enema once to twice as an emergency treatment. Every use of Glycerol Enema should be recorded in the diary timely as required. If laxatives are used, you will need record the name, dosage, time of drug use faithfully in time.

IV. Potential benefits of study participation
You may benefit from this study. The benefits may include improvement of symptoms, even by sham electro-acupuncture treatment. The study may also help doctors and researchers to further evaluate the efficacy of electro-acupuncture treatment for severe functional constipation. The information will be beneficial in the management of other patients with a similar condition in the future.
If you decide to participate in the study, you will get relevant physical and biochemical examination as well the study intervention for free during the study period.

V. Potential side effects, risks, discomforts, and inconveniences
The doctors will make every effort to prevent and treat any side effects brought on by this study.
During acupuncture treatment, you may feel soreness, numbness, heavy, distension sensation, etc., which are normal reactions to acupuncture. Acupuncture treatment may have some adverse effects, but it is rare



Downloaded From: http://annals.org/ by a China Academy of Chinese Medical Sciences User on 09/13/2016

and mild. You may feel fainting due to your individual physique or emotional stress when receive acupuncture needling. Your symptoms should be relieved after the cessation of acupuncture treatment and rest. Bleeding, hematoma, and other phenomena may occur after acupuncture treatment, and these phenomena should disappear after applying local pressure. If infection occurs in the needle site, your doctor will handle it timely.

With the treatment following the study protocol in the study, if you experience adverse reactions and events related to acupuncture treatment, please feel free to call your doctor for help. The doctor will provide you timely treatment. If injuries have been confirmed and are caused by adverse reactions and events of the study, the study group will deal with them appropriately in accordance with relevant provisions.

If you experience any discomfort or new change of your symptoms, or any other unforeseen circumstances during study period, regardless of whether these events is relevant with treatment of the study or not, you shall promptly notify your doctor, and he / she will evaluate the condition and give you appropriate medical treatment.

VI. Payments/compensation for participation

If you participate in the study, during the study, you will get relevant physical and biochemical examination and acupuncture treatment for free.

If adverse events occur during the study, medical experts committee will identify whether they are related to the study. The study group will deal with it appropriately in accordance with relevant provisions.

The treatment and examination required for your concomitant diseases will not be free of charge.

VII. Confidentiality of personal information

All the information related to your participation in this study will be kept confidential by the institute where your participation takes place. Only the institutes responsible for the study, clinical research institutes, and ethics committees may have access to your medical records. Your name will not appear in any publications or reports related to this study.

We will make every effort to protect the privacy of your personal medical information as per legal requirements and laws.

VIII. How to acquire extra information?

You can ask any questions about the study at any time and will get answers timely.

If we notice any new information that may affect your willingness and decision to continue participating in the study, the doctor will keep you informed.

IX. Can you voluntarily choose to participate in or withdraw from the study?

Whether to participate in this study or not entirely depends on your desire. You can refuse to participate in the study, or withdraw from the study at any time during the study, which will not affect the relationship between you and your doctor and will not affect your medical interests or interests in other areas.

For the consideration of your best interests, doctors or researchers may terminate your participation in this study at any time.

If you withdraw from the study for any reason, you may be asked for information related of acupuncture treatment or the use of other medications during your participation of the study. If the doctor considers it necessary, you may also be asked to have some laboratory tests and physical examinations performed.

X. What you need to do now?

Decide whether to participate in this study or not.

Before you make the decision to participate in the study, please ask your doctor if you have any concerns.

Thank you for reading the above information. If you decide to participate in this study, please tell your doctor, he / she will help you make arrangement for the study.

Please keep this document for your own record.



Downloaded From: http://annals.org/ by a China Academy of Chinese Medical Sciences User on 09/13/2016

Informed Consent: Signature Page

Study title: The effectiveness and safety of electro-acupuncture for severe functional constipation- a multicenter randomized controlled trial
Organizer of this study：
Collaborative institute：

Statement of agreement
I have read the above information about this study and have the opportunity to discuss this study with my doctor and ask questions. All my questions were answered satisfactorily.
I understand the potential risks and benefits from participation in this study. I understand the participation of the study is voluntary and I confirm that I was given sufficient time for consideration of study participation. I confirm that I understand that:
I can always ask the doctor for additional/more information.
I can withdraw from the study at any time without discrimination or retaliation and my medical treatment and interests will not be affected.
I understand that if I withdraw from the study, I will tell the doctor the changes of my disease condition and complete the relevant physical and biochemical examinations if needed, which will be very helpful for the whole study.
If I need to take any other medications due to the changes of my medical condition, I will seek medical advice from the doctor beforehand or afterwards tell the doctor truthfully.
I agree to allow the research institute, collaborative institutes, and ethics committees to inspect the data relevant to my study participation.
I will receive a signed and dated copy of the informed consent form.
Finally, I decide and agree to participate in this study and ensure the adherence to doctor's orders to the best I can.

Signature of patient Year month day

Telephone:

I confirm that I have explained this study in detail to the patient, including patient's rights as well as the potential benefits and risks, and have given the patient a signed copy of the informed consent form.

Signature of doctor Year month day

Office phone number of doctor:

**6. Quality Control**
**6.1 Quality Control**
In our trial we will try to avoid selection bias as far as possible by using central randomized controlled design, and also by strictly including the participants according to the clear inclusion and exclusion criteria. Participants and the professional evaluators will be blinded; the statisticians who take care of our statistical analysis will also know nothing about patient assignment.
We have 3 levels of quality control:
Level 1: one or more researchers from each site will be assigned to check the performance of the trial.
Level 2: three or more researcher staff from Guang'anmen Hospital will be assigned to inspect the performance of the trial at all sites.
Level 3: five or more researchers from the Institute of Basic Research in Clinical Medicine, China Academy of Chinese Medical Sciences will be assigned to audit the performance of the trial at all sites.
We will perform the inspection and auditing in December 2012, in the middle of 2013, and in the middle of 2014.
**6.2 Quality Guarantee**



Downloaded From: http://annals.org/ by a China Academy of Chinese Medical Sciences User on 09/13/2016

Before the start of the clinical trial, we will have a clinical training for all the researchers. After the training, the researchers should be familiar with the trial protocol, and the standard operation procedure (SOP) of the trial's performance.

**6.3 Compliance Improvement**

$$\text{Rate of compliance} = \frac{\text{The times of the treatment accepted by the participant actually}}{\text{The times of the treatment according to the protocol}} \times 100\%$$

The rate of compliance no less than 80% will be considered as a good compliance. To improve the participants' compliance, we will take measures as followed:

- Participants should participate the trial of their own accord, and sign the informed consent;
- Researchers should communicate with the participants frequently, patiently, and with concern for getting a better physician-patient relationship, and explain the related examination, interventions, and form filling at length.
- Researchers should record the participants' contact information in detail for the convenience of the follow-up.
- Before the randomization, researchers should inform the participants that all the cost referring to the examination and treatment would be exempted. Participants who complete all 8-week treatment and 12-week follow-up will get a subsidy of 180 RMB.
- The defecation diaries of the screening and treatment periods will be distributed weekly. The diaries of the follow-up period will be distributed every 4 weeks. Assessor should take back the completed diaries in time, and verify the related items with the participants.
- We will afford rescue medicines for the participants for severe constipation.
- We will remind the participants to fill in their defecation diaries in time by affording them brochures, or by texting.
- For participants who have less compliance, we will still follow them up for recording the related outcomes through phone or message.

**6.4 Data and Safety Monitoring Board (DSMB)**

To monitor the data and safety of this study, a DSMB composed by 7 experts will be organized before the starting of the trial. The overall responsibility of the DSMB is to protect the ethical and safety interests of subjects recruited into the project "The Efficacy and Safety Study of Electro-acupuncture for Severe Chronic Functional Constipation - a Multicenter, Randomized Controlled Trial", while protecting as far as possible the scientific validity of the data. The DSMB will have meeting at predefined times as at least twice a year to evaluate primary endpoint, to identify potential treatment harm and all cause mortality/morbidity and to identify potential treatment benefit. The data and safety-monitoring plan of DSMB is in detail in the Appendix.

**7. Data Management**

**7.1 Flow Chart of the Data Management**



Downloaded From: http://annals.org/ by a China Academy of Chinese Medical Sciences User on 09/13/2016

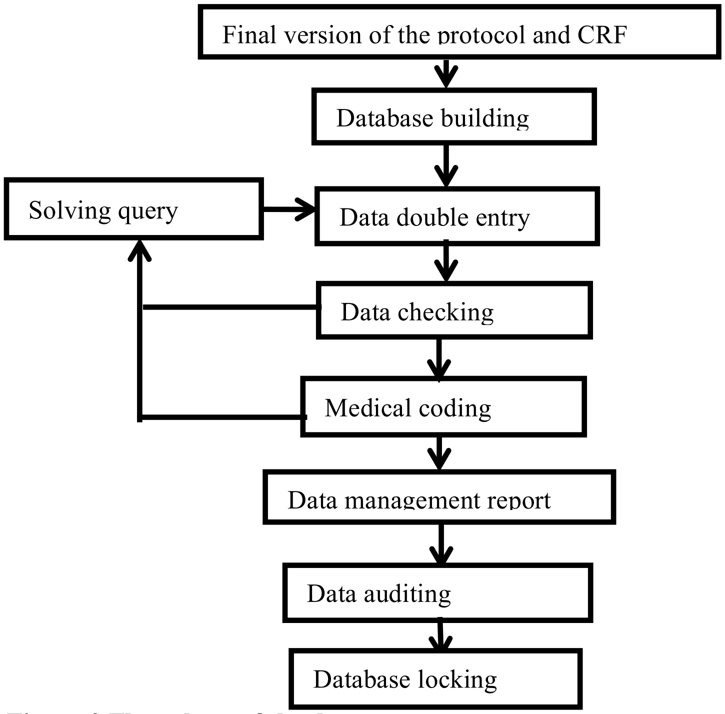


**Figure 3 Flow chart of the data management**

**7.2 The Raw Data Management and Archiving**

We use Remote Data Capture (RDC) system to perform data entry. The research assistants will fill out all the electrical CRF through RDC system. Researchers will inspect the eCRF, and signed electrically for the eCRF going into effect. The eCRF and the trace of eCRF revising will be left in the Oracle database.

**7.3 Data Entry and Storage**

**7.3.1 Database Building and Testing, Data Entry Interface**

The eCRF will be noted through CDISC SDTM standard, and the data entry interface will be generated through the Oracle Clinical software. The data entry interface should be in accordance with the paper-version CRF as far as possible. The inputted data will be stored in the Oracle database.

After preliminarily setting up the database, the entry clerks will input some analog data according to the CRF to test the database. The testing contains: (1) the agreement of the data entry interface and the paper-version CRF; (2) the agreement of the exported data from the database and the analog data; (3) the agreement of the structure of the exported database and the paper-version CRF. After the testing, data administrators should revise the database and make a testing report. Then they electrically signed on the approval page of the database to indicate that the testing is completed. The electrical files of the analog CRF, Noted CRF, screenshot of the data entry interface, database testing report, and the approval page of the database should be saved. If the database updates during the trial, the electrical files mentioned above are also need to be updated.

**7.3.2 Data Entry and Inspection**

The research assistants take charge of the data entry for our trial. Before the entry, all the research assistants will accept the related training according to the data entry handbook. Researchers will inspect the database, and then sign electrically to let the data go in to effect.

**7.4 Data Verification and Problems Solving**

Researchers will verify the data through Data Verification Plan (DVP) approved by the data administrator and the statisticians. Data queries will be inputted to a data query database, and form the DCF. After being inspected, the DCF will then be handed back to the original site, and the researchers of the site should answer the queries. Any revision of the database will be recorded through the RDC software.

**7.5 Medical Coding**

A data administrator who has the medicine background will take charge of the medical coding. The contents of the coding are the clinical history, adverse events, and combined medication. The clinical history and adverse events will be coded through MedDRA dictionary (Version 13.0), and the combined



Downloaded From: http://annals.org/ by a China Academy of Chinese Medical Sciences User on 09/13/2016

medication will be coded via WHO DD dictionary (Version 2007.03). The lead researchers will verify the coded e-files.

**7.6 Data Report**

Data report contains the aspects as followed: (1) members of the project; (2) disagreement from the primary data management plan; (3) actual finish time of every project; (4) problems and the solution during the data management (if have any); (5) reconstruction of the database (if have any); (6) distribution of the participants; (7) participants who disobey the trial protocol; (7) classifying plan of the statistical analysis population.

Data report will be performed monthly since the first entry of the eCRF.

**7.7 Data Auditing and Blinding Review**

When the data checking is finished, a data auditing and blinding review meeting will be hold. On the meeting, the data administrators, statisticians, researchers, clinical inspectors, and other related members would have a discussion on the following items according to the data management report and the data lists:

- Distribution of the participants;
- Protocol disobeying or not;
- Possible outlier;
- Baseline data;
- Outcomes;
- Statistical analysis plan.

Participants will be classified to their suitable statistical analysis sets according to the definition in the protocol. No patient can be excluded from the analysis, unless getting the permission of the meeting participants. All the meeting participants should sign the data locking consent, and the data auditing resolution.

**7.8 Database Locking**

The database will be locked if it fulfills all the aspects as followed:

- All the queries have been solved, and the database has been updated;
- No query has been found through the data inspection;
- The medical coding has been completed;
- The plan of the participants ' classification has been approved;
- The final draft of the SAP has been made, and approved by the project leader.

The leader of the DSMB (Table 1), statisticians and the data administrators will signed the data locking form, and then the database will be locked. The locked database will be sent to the statisticians for further statistical analysis through the data format of SAS.

**8. Data Analysis**

**8.1 Objectives and Hypothesis**

The main objective is to compare the change of mean weekly CSBMs over the 8-week from baseline in the two groups. And we hypothesize that EA is better than SA in increasing CSBMs, relieving the related symptoms of severe constipation, and improving the quality of life.

**8.2 Statistical Analysis Population**

Randomization population will be adopted. According to the intention-to-treat principle, the participants who accept randomization will be included in the randomization population regardless of whether they receive any treatment. Missing data will be handled with multiple imputation methods. The Institute of Basic Research in Clinical Medicine, China Academy of Chinese Medical Sciences and the Biostatistics center in the University of Washington (Seattle) will be responsible for the statistical analysis.

**8.3 Statistical Contents and Methods**

All statistical analyses will be two-sided tests. For all statistical analyses, SAS 9·4 software will be used. A P value of less than 0·05 (α-value of 0·05) is considered to indicate statistical significance.

Analysis of baseline characteristics will analyze the demographic data and other baseline measures. Mean and standard deviation will be used in the continuous variables and percentages in the categorical variables. For comparisons the two independent sample, t-test will be used for continuous variables and Chi-squared test for categorical variables. Non-parametric tests may also be used. A Fisher Exact test will be used to describe the case distribution in the EA group and the SA group and to calculate the total dropout rate and the dropout rate caused by adverse events between the two groups. A Chi-square test is used to analyze the difference in compliance between the two groups to evaluate if the participants in the EA group and the SA group receive the specified treatment according to the protocol.



Downloaded From: http://annals.org/ by a China Academy of Chinese Medical Sciences User on 09/13/2016

As the main objective is to compare the changed number of weekly average CSBMs throughout the 8 weeks of treatment with the average weekly CSBMs during the 2 weeks baseline period, the primary analysis of the patient-level data is the number of CSBMs per week for 8 weeks. It means the total amount of CSBMs during 8 weeks will be divided by eight to achieve the result. (Each patient therefore has one number of weekly CSBMs throughout the 8 weeks of treatment). The null hypothesis is this number of CSBMs is the same between the EA and SA groups, and the alternative hypothesis is that the change from baseline in CSBMs at 8 weeks for the EA group does not equal the SA group. The subjects who do not receive any treatment after randomization or receive treatment but not any efficacy assessment data are classed as missing data, and multiple imputation methods will be used to deal with the missing data. An analysis of covariance (ANCOVA) will be used group, study site and baseline CSBMs will be included in the ANCOVA model. The secondary outcome measures include the following seven items: 1) the proportion of participants with three or more mean weekly CSBMs during weeks 1–8 and weeks 9–20; 2) the change from baseline in mean weekly CSBMs during weeks 9–20; 3) the change from baseline in mean weekly spontaneous bowel movements (SBMs); 4) the change from baseline in mean stool consistency over weeks 1–8; 5) the change from baseline in mean straining scores over weeks 1–8; 6) the change from baseline in PAC-QOL scores at week 8; 7) the proportion and mean weekly frequency of rescue medicine and other defecation assistances used. Chi-square test was used to compare the proportion of participants having three or more CSBMs and the proportion of other rescue medicine and other defecation assistances used between two groups.  Student's t test was used to compare the change from baseline of mean weekly SBMs and the scores in PAC-QOL, stool consistency, and straining between groups. Wilcoxon rank sum test was used to compare the mean weekly frequency of other rescue medicine and other defecation assistances used between groups. Subgroup analysis will be conducted according to the subject's age (<65 years and ≥65 years). All adverse events and serious adverse events will be listed. Poisson regression model will be used to compare the incidence of adverse events between the EA and SA groups.

**9 Ethical principle**

For every study site, only when the trial protocol is approved by the IRB, the enrollment of participant will begin, but all should be after Sep 1, 2012.

**10 Funding**

This study is supported and funded by the program of "the 12th Five-year'' National Science and Technology Pillar Program (2012BAI24B01) by the Ministry of Science and Technology of the People's Republic of China.

**11 References**

1 Stewart WF, Liberman JN, Sandler RS, Woods MS, Stemhagen A, Chee E, Lipton RB, Farup CE: Epidemiology of Constipation (EPOC) study in the United States: relation of clinical subtypes to sociodemographic features. *Am J Gastroenterol* 1999; **94**: 3530–40.

2 Lembo A, Camilleri M. Chronic constipation. *N Engl J Med* 2003; **349**: 1360–68.

3 Camilleri M, Kerstens R, Rykx A, Vandeplassche L: A placebo-controlled trial of prucalopride for severe chronic constipateion. *N Engl J Med* 2008; **358**: 2344–54.

4 Johanson JF, Kralstein J. Chronic constipation: a survey of the patient perspective. *Aliment Pharmacol Ther* 2007; **25**: 599–608.

5 Du WF, Yu L, Yan XK, Wang FC. Meta-analysis on randomized controlled clinical tirals of acupuncture and moxibustion on constipation (Chinese). *Zhongguo Zhen Jiu* 2012; **32**: 92–6.

6 Wang CW, He HB, Li N, Wen Q, Liu ZS: Observation on therapeutic effect of electroacupuncture at Tianshu (ST25) with deep needling technique on functional constipation (Chinese). *Zhongguo Zhen Jiu* 2010; **30**: 705–8.

7 Wang CW, Li N, He HB, Lü JQ, Liu ZS. Effect of electroacupuncture of Tianshu (ST25) on the rational symptoms of functional constipation patients and evaluation on its efficacy satisfaction: a single-center, prospective, practical and randomized control trial (Chinese). *Zhen Ci Yan Jiu* 2010; **35**: 375–9.

8 Rome Foundation. Rome III Diagnostic Criteria for Functional Gastrointestinal Disorders. 2006; http://www.romecriteria.org/criteria/

9 Johanson JF, Wald A, Tougas G, et al. Effect of tegaserod in chronic constipation: a randomized, double-blind, controlled trial. *Clin Gastroenterol Hepatol* 2004; **2**: 796–805.

10 Lin SR, Ke MY, Luo JY, et al. A randomized, double-blind, placebo-controlled trial assessing the efficacy and safety of tegaserod in Participants from China with chronic constipation. *World J*



Downloaded From: http://annals.org/ by a China Academy of Chinese Medical Sciences User on 09/13/2016

*Gastroenterol* 2007; **13**: 732–739.
11 Lewis SJ, Heaton KW. Stool form scale as a useful guide to intestinal transit time. *Scand J Gastroenterol* 1997; **32**: 920–924.
12 Marquis P, De La Loge C, Dubois D, McDermott A, Chassany O. Development and validation of the Patient Assessment of Constipation Quality of Life questionnaire. *Scand J Gastroenterol* 2005; **40**: 540-51.
13 Zhao Z. *A psychometric evaluation of the Chinese version of Patient Assessment of Constipation Quality of Life questionnaire and analysis of influencing factors on quality of life* [Master]. Nanjing, Nanjing Medical University; 2011
14 WHO Regional Office for the Western Pacific. WHO Standard Acupuncture Point Locations in the Western Pacific Region. Manila, Philippines. 2008.

**12 Updates on the original protocol**

As compared to the published protocol (Liu Z, Liu J, Zhao Y, et al. The efficacy and safety study of electro-acupuncture for severe chronic functional constipation: study protocol for a multicenter, randomized, controlled trial. Trials 2013;14:7), the present finalized study protocol had corrected two previous errors and made two minor amendments.

I. The two errors corrected in the finalized study protocol

a) Depth of needle insertion was 30 to 70mm rather than 30 to 80mm in the EA group as the length of the needle body in this trial is 75mm or 50mm (80mm is greater than the needle body length of 75mm). The following was the change: 'Performance: After disinfection, with the patient supine, 0.30 mm × 50 mm or 0.35 mm × 75 mm needles were inserted into ST25 and SP14 slowly and vertically, without manipulation, for approximately 30 to 70 mm until they pierced the muscle layer of the abdominal wall.' The original state was 'needles of the size of 0.30mm×50mm, or 0.35mm×75mm will be inserted into ST25 and SP14 slowly and vertically, without manipulation, for approximately 3 to 8 cm until touching the parietal peritoneum. The standard for reaching the parietal peritoneum is that the participant feels sharp pain again (after the first pain of piercing the skin) and, meanwhile, the manipulator feels resistance from the needle tip'

b) Depth of needle insertion in the SA group was 3 to 5mm rather than 10 to 15mm. A depth of 10 to 15mm is commonly used in real acupuncture practice rather than sham acupuncture settings, and a depth of 3 to 5mm was actually used in the present study. The following was the changes: the depth of needle insertion was 3 to 5mm; the original depth was 1 to 1.5cm.

II. Two amendments were made because of practicality and/or safety concerns; these two amendments were unlikely to alter the study results or conclusion:

a) The exclusion criterion of 'pelvic floor dyssynergia or rectal evacuation disorder' was deleted because of difficulties in diagnosis and irrelevance to study purposes.

b) Information regarding acupuncture procedure in the EA group was changed from using parietal peritoneum as guidance to using the muscle layer of the abdominal wall as guidance (please see depth of needle insertion correction in the EA group above for details). The reason for this change is practicality and safety concerns.



Downloaded From: http://annals.org/ by a China Academy of Chinese Medical Sciences User on 09/13/2016

**Appendix—DSMB for Data and Safety Monitoring Plan (DSMP)**

**1. Data and Safety Monitoring Board**

| Name | Affiliation |
|---|---|
| Lixing Lao, Ph.D., MB | School of Chinese Medicine, The University of Hong Kong |
| Claudia M. Witt, MD, MBA | Institute for Social Medicine, Epidemiology and Health Economics; Charité Universitätsmedizin Berlin |
| Justin C.Y. Wu, MD | Faculty of Medicine, The Chinese University of Hong Kong |
| Hugh MacPherson, Ph. D | Department of Health Sciences, University of York |
| Zhaoxiang Bian, MD, Ph. D | Clinical Division, School of Chinese Medicine, Hong Kong Baptist University |
| Yao Chen, Ph.D | Medical Statistics Research Office, the First Hospital of Peking University |
| Zhiwei Xia, MD | Gastroenterology Department, Peking University Third Hospital |

**Table 1 Members of the data and safety monitoring board.**

**2. Study overview**

Before the starting of the trial, it is planned that in August, 2012, DSMB will review the DSMB Charter, and making recommendations for changes to the trial protocol, as well as all the research sites, qualification of investigators, research purpose, technical roadmap, subject benefit and safety, adverse event reporting procedure, data collection methods, safety/efficacy monitoring procedures, the necessary of maintaining or breaking the blind in the course of reviewing the results, stopping rules for efficacy/safety.

**3. Study process and status monitoring**

Subsequently, the first data review meeting will be held when 1/3 subjects have been randomized and completed 8 weeks treatment. Thereafter, the frequency of scheduled meetings depends on subject enrolment, information accumulated and safety event rates but will occur no less than once a year. Where possible the meetings will be scheduled at a day and time convenient to the members of the DSMB. A schedule of subsequent meetings will be determined by the DSMB.

In the beginning of 2013, a teleconference meeting is planned to collect the DSMB members for overview the work of the first study year. In the middle of the trial, the interim analysis is planned in August 2013, in which DSMB will review and query the progress of the study, adverse event reporting, server adverse event occurrence, quality control condition, data reliability. The independent statistician will submit the report to DSMB.

**4. Data management**

When the trial finished, DSMB will review the performance of trial, adverse event reporting, server adverse event occurrence, quality control condition, original data, data recording, data accreditation, data locking, and the independent statistician will submit the report to DSMB.



Downloaded From: http://annals.org/ by a China Academy of Chinese Medical Sciences User on 09/13/2016
