# Supplementary material for: Factors related to acupuncture response in patients with chronic severe functional constipation: Secondary analysis of a randomized controlled trial
Source: PLoS One. 2017 Nov 22;12(11):e0187723. doi: 10.1371/journal.pone.0187723 (PMC5699843; doi:10.1371/journal.pone.0187723)
Supplement: S2 Text — (PDF) [file pone.0187723.s002.pdf]

Ethical Approvals of all local ethical committees

Guang An Men Hospital is the leader hospital and the trial was conducted in 15 hospitals. The protocol was submitted to the Ethical committee of Guang An Men Hospital firstly and got approved. Then the protocol was submitted to the ethical committees of all the other 14 hospitals where the trial taking place and all got approved.

Ethical Approvals of Guang An Men Hospital

Human Research Ethics Committee Approval

By Human Research Subject Ethics Committee of Guang An Men Hospital, CACMS

No. 2012-092

No. 2012-092 Project		The Efficacy and Safety Study of Electro-acupuncture for Severe Chronic Functional Constipation - a Multicenter, Randomized Controlled Trial	
Sponsor		the 12th National Key Technology Support Program of the Ministry of Science and Technology of the People's Republic of China	
Project No.	2012BAI24B01	PI	Liu Baoyan; Liu Zhishun
Participating Centers		Beijing: Guang'anmen Hospital, Beijing TCM Hospital, Huguosi TCM hospital, 301 Hospital, the 3rd Hospital of Zhejiang University of TCM, Dongzhimen Hospita l; Chengdu: West China Hospital of Sichuan University; Hefei: An'hui TCM Hospital; Nanjing: Jiangsu TCM Hospital, Nanjing University of TCM; Guangzhou: Guangdong TCM hospital; Wuhan: Wuhan Hospital of Integrated TCM and Western Medicine; Harbin: Heilongjiang Academy of TCM; Tianjin: the 1st Hospital of Tianjin University of TCM; Shanghai: Shanghai Yueyang Hospital.	

Ethicality	
Comments	Vote
1. Agree	6
2. Agree after the revising	0
3. Disagree	0

Manning	
Comments	Vote
1. Agree	6
2. Agree after the revising	0
3. Disagree	0

Protocol	
Comments	Vote
1. Agree	6
2. Agree after the revising	0
3. Disagree	0

Inform Consent Form	
Comments	Vote
1. Agree	6
2. Agree after the revising	0
3. Disagree	0

Conclusion	
Comments	Chair Signature
1. Agree √	Zhang Yunru
2. Agree after the revising	
3. Disagree	

**Human Research Subject Ethics Committee Member Signature** Name	Gender	Specialty	Signature
Zhang Yunru	Female	TCM Internal Medicine	Zhang Yunru
Tong Xiaolin	Male	TCM Hemadenology	leave of absence
Yang Herong	Female	Science of Law	Yang Herong
Piao Bingkui	Male	TCM oncology	leave of absence
Gu Lizhen	Female	Pharmacology/Toxicology	Gu Lizhen
Wang Yinghui	Male	Acupuncture	Wang Yinghui
Shen Ruiying	Female	Medical ethics	Shen Ruiying
Zhao Jun	Male	Psychology	Zhao Jun

中国中医科学院广安门医院科研课题
伦理委员会综合审查意见表

中国中医科学院广安门医院医学伦理委员会 2012 第 092 号

课题名称	电针治疗严重功能性便秘有效性和安全性多中心随机对照试验		
课题级别	“十二五”国家科技支撑计划		
课题批准号	2012BAI24B01	课题负责人	刘志顺
临床协作单位	中国中医科学院广安门医院、北京中医医院、护国寺中医院、301 医院、东直门医院、安徽省中医院、上海中医药大学岳阳中西医结合医院、四川大学华西医院、浙江中医药大学附属第三医院、江苏省中医院、南京中医药大学、广东省中医院、武汉市中西医结合医院、天津中医药大学一附院、黑龙江中医研究院		

对课题立题伦理性的审核	
意见	委员投票数
1. 同意	6
2. 作必要的修正后同意	0
3. 不同意	0

对研究者、人员配备的审核	
意见	委员投票数
1. 同意	6
2. 作必要的修正后同意	0
3. 不同意	0

对临床研究方案的审核	
意见	委员投票数
1. 同意	6
2. 作必要的修正后同意	0
3. 不同意	0

对知情同意书的审核	
意见	委员投票数
1. 同意	6
2. 作必要的修正后同意	0
3. 不同意	0

综合审核意见：

1. 同意	✓	主任委员签名：张云亭
2. 作必要的修正后同意		
3. 不同意		

日期：2012.9.6

本次伦理委员会出席会议的委员情况

项目名称：电针治疗严重功能性便秘有效性和安全性多中心随机对照试验（”十二五”国家科技支撑计划）

第 一 次会议

姓名	性别	专业基本情况	签名
张云如	女	中医内科	张云如
仝小林	男	中医内分泌	请假
杨荷蓉	女	法学	杨荷蓉
朴炳奎	男	中西医结合肿瘤	请假
顾丽贞	女	药理毒理	顾丽贞
王映辉	男	针灸科研及临床	王映辉
沈瑞英	女	医学伦理学	沈瑞英
赵军	男	心理学	赵军

日期：2012.9.6

1. Guang An Men Hospital of China Academy of Chinese Medical Sciences (South Branch

Approved By Human Research Subject Ethics Committee of Guang An Men Hospital, CACMS No. 2012-092; approved by Guang An Men Hospital (South branch), CACMS

No. 2012-092 Project		The Efficacy and Safety Study of Electro-acupuncture for Severe Chronic Functional Constipation - a Multicenter, Randomized Controlled Trial	
Sponsor		the 12th National Key Technology Support Program of the Ministry of Science and Technology of the People's Republic of China	
Project No.	2012BAI24B01	PI	Liu Baoyan; Liu Zhishun
Participating Centers		Beijing: Guang'anmen Hospital, Beijing TCM Hospital, Huguosi TCM hospital, 301 Hospital, the 3rd Hospital of Zhejiang University of TCM, Dongzhimen Hospita l; Chengdu: West China Hospital of Sichuan University; Hefei: An'hui TCM Hospital; Nanjing: Jiangsu TCM Hospital, Nanjing University of TCM; Guangzhou: Guangdong TCM hospital; Wuhan: Wuhan Hospital of Integrated TCM and Western Medicine; Harbin: Heilongjiang Academy of TCM; Tianjin: the 1st Hospital of Tianjin University of TCM; Shanghai: Shanghai Yueyang Hospital.	

Ethicality	
Comments	Vote
1. Agree	6
2. Agree after the revising	0
3. Disagree	0

Manning	
Comments	Vote
1. Agree	6
2. Agree after the revising	0
3. Disagree	0

Protocol	
Comments	Vote
1. Agree	6
2. Agree after the revising	0
3. Disagree	0

Inform Consent Form	
Comments	Vote
1. Agree	6
2. Agree after the revising	0
3. Disagree	0

Conclusion	
Comments	Chair Signature
1. Agree √	Zhang Yunru
2. Agree after the revising	
3. Disagree	

中国中医科学院广安门医院科研课题
伦理委员会综合审查意见表

中国中医科学院广安门医院医学伦理委员会 2012 第 092 号

课题名称	电针治疗严重功能性便秘有效性和安全性多中心随机对照试验		
课题级别	“十二五”国家科技支撑计划		
课题批准号	2012BAI24B01	课题负责人	刘志顺
临床协作单位	中国中医科学院广安门医院、北京中医医院、护国寺中医院、301 医院、东直门医院、安徽省中医院、上海中医药大学岳阳中西医结合医院、四川大学华西医院、浙江中医药大学附属第三医院、江苏省中医院、南京中医药大学、广东省中医院、武汉市中西医结合医院、天津中医药大学一附院、黑龙江中医研究院		

对课题立题伦理性的审核	
意见	委员投票数
1. 同意	6
2. 作必要的修正后同意	0
3. 不同意	0

对研究者、人员配备的审核	
意见	委员投票数
1. 同意	6
2. 作必要的修正后同意	0
3. 不同意	0

对临床研究方案的审核	
意见	委员投票数
1. 同意	6
2. 作必要的修正后同意	0
3. 不同意	0

对知情同意书的审核	
意见	委员投票数
1. 同意	6
2. 作必要的修正后同意	0
3. 不同意	0

综合审核意见：

1. 同意	✓	主任委员签名：张之文
2. 作必要的修正后同意		
3. 不同意		

日期：2012.9.6

同意

2012.10.23

2. West China Hospital of Sichuan University
NO. 2012-211

Department: Department of Integrated western medicine and TCM	Sub-site PI: Dr Li Ning (assistant chief physician)	
Trial name	The Efficacy and Safety Study of Electro-acupuncture for Severe Chronic Functional Constipation - a Multicenter, Randomized Controlled Trial	
Protocol	Version: N/A	Date: Sep 1 2012
ICF	Version: N/A	Version: N/A

Comment:
1. The investigators qualified for ethical requirements.
2. The protocol and ICF qualified for ethical requirements.

Conclusion:
■ Approved
☐ Approved after revision
☐ Re-review after revision
☐ Not approved
☐ Terminated/paused

Please follow the relevant laws, regulations and rules (the SFDA's <Drug clinical trial quality management standard"(2003), <Medical instrument clinical trial regulations > (2004), WMA < Declaration of Helsinki> and CIOMS <Human body biomedical research international ethical guidelines>, the ministry of health <Biomedical research involving human neighborhood review method (trial) (2007)), follow the protocol and ICF approved by ethics committee to conduct clinical trials, protect the health of the subjects and power.
During the experiment, if there is any change about the PI, the clinical study scheme, ICF, etc., the applicant should submit the revised protocol for re-review.
If there is any serious adverse events occurs, the applicant should submit the serious adverse event reports; After emergency report, details of the follow-up report should be reported in serious adverse events report as soon as possible.
Please submit the annual inspection and regular tracking report. When any situation which may significantly impact tests or increase the risk of the subjects, the applicant should submit a written report to the ethics committee timely.
If there is any participant who did not qualified for the inclusion criteria or qualified for the exclusion, giving wrong therapy or dose, giving solutions such as follow on the prohibited drug combination of situation or any other situation which may affect the rights and interests of subjects, thus the applicant/monitor/investigator should submit the report.
If the applicant will terminate or pause the clinical trials, please submit a pause/suspend report. The summary report need to be submitted after completing the clinical trial.

四川大学华西医院临床试验与生物医学伦理专委会审查批件

2012年 审（211）号

科室（专业）： 中西医结合科	项目负责人姓名及职称： 李宁 副主任医师	
项目名称	电针治疗严重功能性便秘有效性和安全性国际多中心随机对照试验	
研究方案	版本号：无	版本日期：2012.9.1
知情同意书	版本号：无	版本日期：无

审查意见：

1. 研究者资质符合伦理要求。
2. 研究方案及知情同意书基本符合伦理要求。

审查结果：■同意 □作必要修正后同意 □修正后再审 □不同意 □终止或暂停

请遵循我国相关法律、法规和规章（SFDA《药物临床试验质量管理规范》（2003）、《医疗器械临床试验规定》（2004）、WMA《赫尔辛基宣言》和CIOMS《人体生物医学研究国际道德指南》、卫生部《涉及人的生物医学研究伦理审查办法（试行）（2007）》），遵循伦理委员会批准的方案和知情同意书开展临床试验(研究),保护受试者的健康与权利。

在试验（研究）过程中，若变更主要研究者，对临床研究方案、知情同意书等的任何修改，请申请人提交修正案审查申请。

发生严重不良事件，请申请人及时提交严重不良事件报告；紧急报告之后，尽快提交详细的严重不良事件随访报告。

请递交年度和定期跟踪审查报告；当出现任何可能显著影响试验（研究）进行或增加受试者危险的情况时，请申请人及时向伦理专委会提交书面报告。

试验（研究）纳入了不符合纳入标准或符合排除标准的受试者，符合中止试验（研究）规定而未让受试者退出试验（研究），给予错误治疗或剂量，给予方案禁止的合并用药等没有遵从方案开展研究的情况；或可能对受试者的权益/健康、以及研究的科学性造成不良影响等违背伦理原则与规范的情况，请申办者/监查员/研究者提交违背方案报告。

申请人暂停或提前终止临床试验（研究），请及时提交暂停/终止试验（研究）报告。完成临床试验（研究），请申请人提交结题报告。

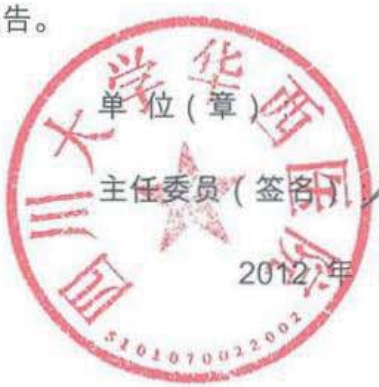

单位（章）

主任委员（签名）

2012年 10月10日

3. Guangdong Hospital of TCM
No. AF/04-05/10.0

Date	September 7, 2012
Address	No 11 meeting room, Guangdong TCM Hospital<br>No 111, Dade Road, Guangzhou, Guangdong Province, China
Clinical research approval	N/A
Trial title	The Efficacy and Safety Study of Electro-acupuncture for Severe Chronic Functional Constipation - a Multicenter, Randomized Controlled Trial
Document	1. Trail assignment(2012BAI24B00)<br>2. Protocol (V. 2012BAI24B00/July 21 2012)<br>3. CRF (V. 2012BAI24B00/July 21 2012)<br>4. Investigator handbook (V 1.0/August 2012)<br>5. Investigator list, PI CV<br>6. Participant recruitment<br>7. Brief introduction of the trail (V 1.0/August 2012)<br>8. ICF (V 1.0/August 2012)<br>9. Technical cooperation contract
Applicant	Ministry of Science and Technology<br>State administration of TCM
Research site	Guangdong TCM Hospital
Site PI	Dr Fu Wenbin
Method of review	Conference review
Committee member	Liu Jun, Li Yan, Xia Ping, Chang Gang, Yang Jinghua, Li Likai, Yu Yijun
Comment	According to the SFDA's <Drug clinical trial quality management standard"(2003), the ministry of health <Biomedical research involving human neighborhood review method (trial) (2007)), WMA < Declaration of Helsinki> and CIOMS <Human body biomedical research international ethical guidelines>, the trial of < The Efficacy and Safety Study of Electro-acupuncture for Severe Chronic Functional Constipation - a Multicenter, Randomized Controlled Trial > was approved by our ethical committee.
Declaration	This approval will be put on records at our ethical committee. Please contact us if you have any different comment about the feasibility.<br>If the applicant will terminate or pause the clinical trials, please submit a pause/suspend report.<br>If there is any serious adverse events occurs, the applicant should submit the serious adverse event reports; After emergency report, details of the follow-up report should be

	reported in serious adverse events report as soon as possible.<br>During the experiment, if there is any change about the PI, the clinical study scheme, ICF, etc., the applicant should submit the revised protocol for re-review.<br>The summary report need to be submitted after completing the clinical trial 1 month before the deadline of this approval.		
Validity	Sep 7 2012 -<br>Sep 7 2015	Re-review<br>Anticipated date	1 year<br>Sep 7 2013
Contact	0086-020-81887233-30818 Dr He Tinghui		
Signature of chief	Liu Yang		
Stamp of committee			
Date	Sep 7 2012		

广东省中医院伦理委员会
Institutional Ethics Committee of Guangdong Provincial Hospital of Traditional Chinese Medicine
伦理审查批件
Approval Notice

批件号：广东省中医院伦理委员会 B2012-57-01

审查会议日期	2012 年 09 月 07 日		
审查会议地点	广东省广州市大德路 111 号广东省中医院东区 11 楼会议室		
临床研究批文			
临床研究项目	电针治疗严重功能性便秘有效性和安全性国际多中心随机对照试验		
审查文件	1. 课题任务书（2012BAI24B00）<br>2. 临床研究方案，版本号：2012BAI24B00/2012.7.21<br>3. 临床试验观察表，版本号：2012BAI24B00/2012.7.21<br>4. 研究者手册，版本号：第一版/2012.8.15<br>5. 课题组人员名单，课题负责人履历<br>6. 招募受试者的材料<br>7. 向受试者提供的研究简介，版本号： 1.0 版/2012.8.15<br>8. 向受试者提供的知情同意书，版本号：1.0 版/2012.8.15<br>9. 技术合作合同		
申办者	国家科技部、国家中医药管理局		
临床研究单位	广东省中医院		
主要研究者	符文彬教授		
伦理审查方式	会议审查		
参会委员	刘军、李艳、夏萍、常锦、杨京华、李立凯、余谊君		
审查意见	根据中华人民共和国国家药品食品监督管理局 2003 年颁布实施的《药物临床试验质量管理规范》、卫生部 2007 年颁布的《涉及人的生物医学研究伦理审查办法》、《赫尔辛基宣言》、国际医学科学组织委员会颁布的《人体生物医学研究国际道德指南》的伦理原则，经本伦理委员会审查，同意按照上述临床研究方案和上述已通过审查的文件进行电针治疗严重功能性便秘有效性和安全性国际多中心随机对照试验项目的临床研究。		
伦理委员会声明	本批件将在各中心机构及其伦理委员会备案。如果对方案在本机构的可行性（包括研究者的资格与经验、设备与条件等）有不同意见，请及时与本伦理委员会联系。<br>如项目暂停/提前终止/完成临床研究，请及时通知伦理委员会。如发生严重不良事件以及影响研究风险受益比的非预期不良事件，应及时报告本伦理委员会，如临床研究方案、知情同意书的任何修改，主要研究者更换，应及时通知伦理委员会，重新审查，获得批准后执行。发现影响受试者参加研究意愿的违反方案情况应及时报告，同时，请在本批件失效日期前 1 个月提交研究进度/结题报告，以便对该项目进行跟踪审查。		
批件有效期	自 2012 年 09 月 07 日起<br>至 2013 年 09 月 07 日止	跟踪审查频率<br>预计审查日期	1 年<br>2013 年 09 月 07 日
联系电话	020-81887233 转 30818，联系人：何庭辉		
主任委员签字	刘军		
		广东省中医院伦理委员会（盖章）	
		日期：2012 年 09 月 07 日	

4. Heilongjiang Institute of TCM

No 2012-11

No. 2012-092 Project		The Efficacy and Safety Study of Electro-acupuncture for Severe Chronic Functional Constipation - a Multicenter, Randomized Controlled Trial	
Sponsor		the 12th National Key Technology Support Program of the Ministry of Science and Technology of the People's Republic of China	
Project No.	2012BAI24B01	Site PI	Wang Shun
Participating Centers		Beijing: Guang'anmen Hospital, Beijing TCM Hospital, Huguosi TCM hospital, 301 Hospital, the 3rd Hospital of Zhejiang University of TCM, Dongzhimen Hospita l; Chengdu: West China Hospital of Sichuan University; Hefei: An'hui TCM Hospital; Nanjing: Jiangsu TCM Hospital, Nanjing University of TCM; Guangzhou: Guangdong TCM hospital; Wuhan: Wuhan Hospital of Integrated TCM and Western Medicine; Harbin: Heilongjiang Academy of TCM; Tianjin: the 1st Hospital of Tianjin University of TCM; Shanghai: Shanghai Yueyang Hospital.	

Ethicality	
Comments	Vote
1. Agree	6
2. Agree after the revising	0
3. Disagree	0

Manning	
Comments	Vote
1. Agree	6
2. Agree after the revising	0
3. Disagree	0

Protocol	
Comments	Vote
1. Agree	6
2. Agree after the revising	0
3. Disagree	0

Inform Consent Form	
Comments	Vote
1. Agree	6

2. Agree after the revising	0
3. Disagree	0

Conclusion	
Comments	Chair Signature
1. Agree √	Bai Xiu yun
2. Agree after the revising	
3. Disagree	

Date: Sep 12, 2012

黑龙江省中医医院科研课题伦理委员会综合审查意见表

黑龙江省中医医院医学伦理委员会 2012 第 11 号

课题名称	电针治疗严重功能性便秘有效性和安全性多中心随机对照试验		
课题级别	“十二五”国家科技支撑计划		
课题批准号	2012BAI24B01	课题负责人	王　顺
临床协作单位	中国中医科学院广安门医院、北京中医医院、护国寺中医院、301 医院、东直门医院、安徽省中医院、上海中医药大学岳阳中西医结合医院、四川大学华西医院、浙江中医药大学附属第三医院、江苏省中医院、南京中医药大学、广东省中医院、武汉市中西医结合医院、天津中医药大学一附院、黑龙江省中医医院		

对课题立题伦理性的审核	
意　见	委员投票数
1、同意	6
2、作必要的修正后同意	0
3、不同意	0

对研究者、人员配备的的审核	
意　见	委员投票数
1、同意	6
2、作必要的修正后同意	0
3、不同意	0

对临床研究方案的审核	
意　见	委员投票数
1、同意	6
2、作必要的修正后同意	0
3、不同意	0

对知情同意书的审核	
意　见	委员投票数
1、同意	6
2、作必要的修正后同意	0
3、不同意	0

综合审核意见

1、同意	✓	主任委员签名：白秀雪
2、作必要的修正后同意		
3、不同意		

日期：2012.09.12

5. Beijing Hospital of TCM
No 201222

Trial Title	The Efficacy and Safety Study of Electro-acupuncture for Severe Chronic Functional Constipation - a Multicenter, Randomized Controlled Trial	
Sponsor	the 12th National Key Technology Support Program of the Ministry of Science and Technology of the People's Republic of China	
Project No.	2012BAI24B01	
Non-drug treatment study	Yes ■ No □	
Group	Treatment group	Control Group
Intervention	Points:Bilateral Tianshu (ST25) Fujie(SP14),Shangjuxu (ST37). Electric stimulator is applied to bilateral ST25 and SP14. Every session will last for 30 minutes per day. The participants are treated continuously for 8 weeks. During the first 2 weeks, 5 sessions will be given per week, and 3 sessions per week in the rest 6 weeks.	Points:Sham Bilateral Tianshu (ST25), Fujie(SP14), Shangjuxu (ST37). Electric stimulator is applied to bilateral ST25 and SP14 with no current output. Treatment duration and frequency is the same as treatment group.
Key Operation	Point location: According to China national standard 2006<GB/T 12346-2006 Nomenclature and location of acupuncture point>. Performance: After sterilizing on the acupuncture skin, With the patient supine, 0.30 mm × 50 mm or 0.35 mm × 75 mm needles were inserted into ST25 and SP14 slowly and vertically, without manipulation, for approximately 30 to 70 mm until they pierced the muscle layer of the abdominal wall. Electric stimulator is applied to bilateral ST25 and SP14 with dilatational wave, 10/50 Hz and electric current between 0.1 and 1.0 mA. The participant's abdominal muscle twitching mildly is the	Sham points location are: about 2cm away from ST25, middle of Spleen and Stomach Channel; about 3cm from SP14, middle of Spleen and Stomach Channel; one point beside ST37, middle of Stomach and Gallbladder Channel. Performance: The needle is inserted after sterilizing on the skin by 0.3 to 0.5cm until the needle can be vertically fixed on the skin. No twirling lifting and thrusting manipulation. The sham electric stimulator is applied to bilateral sham ST25 and sham SP14 with dilatational wave, 10/50 Hz and electric current 0.5 mA. The metal wire has been cut off inside with a same outlook as

	appropriate dose. Bilateral ST37 are given inserting of 3cm, twirling lifting and thrusting for 3 times. Local sour and heavy feeling is appropriate dose. Steady small twirling lifting and thrusting manipulation will be operated for 3 times in one session.		the real electric stimulator, which has no current output. Length of Treatment and the treatment sessions are the same as treatment group.
Duration	The research period is 22 weeks: 2 weeks screening, 8 weeks for treatment, 12 weeks for follow-up.		The research period is 22 weeks: 2 weeks screening, 8 weeks for treatment, 12 weeks for follow-up.
Sample size	40		40
Equipment	SDZ-V electro-acupuncture apparatus		Sham SDZ-V electro-acupuncture apparatus
Manufacture factory	Huatuo, made in Suzhou, China		Huatuo, made in Suzhou, China
Direction	See key operation		See key operation
PI Hospital	Guang An Men Hospital of CACMS		
Site department	Acupuncture center	Site chief	Dr Wang Linpeng
Site PI	Dr Wang Linpneg		
Site name		Site chief	
1	Beijing TCM Hospital	Dr Wang Linpeng	
Document	1. Protocol (V. August 28 2012)<br>2. PI CV<br>3. Ethical review application form<br>4. ICF(V. August 28 2012)<br>5. CRF		
Trial design	■ Single blinded controlled<br>☐ Double blinded controlled<br>☐ No blinded controlled<br>☐ No controlled		
Investigator qualification	■ Qualified	☐ Not Qualified	
Manning	■ Qualified	☐ Not Qualified	
Research aim	■ Proper	☐ Not Proper	
Research condition	■ Qualified	☐ Not Qualified	
Participant recruitment	■ Reasonable	Not Reasonable	
Document for Participant (Easy to understand)	■ Yes	☐ No	
ICF	■ Proper	☐ Not Proper	
Did the advantage more than disadvantage for participants?	■ Yes	☐ No	
Is there any effect treatment or	■ Yes	☐ No	

emergency treatment if any AE related to the trial happens to participants?	

Comment

Committee member anticipated: 9<br>Present: 6<br>Absent: 3	
Summary	
Comment	Vote
1. Approved	6
2. Approved after revision	
3. Not approved	
4. Terminated/Paused	
Other comment or advice:<br>1. If there is any serious adverse events occurs, the applicant should submit the serious adverse event reports; After emergency report, details of the follow-up report should be reported in serious adverse events report as soon as possible.<br>2. During the experiment, if there is any change about the PI, the clinical study scheme, ICF, etc., the applicant should submit the revised protocol for re-review.<br>3. The summary report need to be submitted after completing the clinical trial.<br>4. Since the date approved, annual report should be submitted 1 month before the review date.	
Conclusion:<br>According to the SFDA's <Drug clinical trial quality management standard"(2003), WMA < Declaration of Helsinki>, CIOMS <Human body biomedical research international ethical guidelines>, the trial was approved by our ethical committee.	

首都医科大学附属北京中医医院医学伦理委员会

科研临床研究（非药物）会议审评意见

京中医（伦）审批号：201222

科研项目名称	电针治疗严重功能性便秘有效性和安全性国际多中心随机对照试验	
项目来源	“十二五”国家科技支撑计划项目	
课题任务书编号	2012BAI24B01	
非药物治疗研究	是☒ 否 □	
分 组	试验组	对照组
治疗方案简述	取穴：双侧天枢、腹结、上巨虚。双侧天枢和腹结穴加电针。<br>每次留针 30 分钟，前两周治疗 5 次/周，后 6 周治疗 3 次/周，连续治疗 8 周，共治疗 28 次。	取穴：双侧天枢旁、腹结旁、上巨虚旁非穴点。双侧天枢旁和腹结旁非穴点针柄上接电针，但实际未通电；频次、疗程同试验组。
操作要点	穴位定位：参照 2006 年中华人民共和国国家标准（GB/T 12346-2006）《腧穴名称与定位》<br>操作方法：仰卧位，皮肤常规消毒。天枢和腹结穴，采用 2～3 寸不锈钢毫针快速破皮，然后缓慢垂直深刺，直至腹膜壁层即止（刺至腹膜壁层的标准：患者针刺破皮痛后再次感觉撅痛或较剧烈的刺痛，同时医者自觉针尖抵触感），不提插捻转，再分别横向连接电针仪电极于双侧天枢和腹结穴的针柄上。电针参数：疏密波，2/15Hz、电流强度 0.1ma-1.0ma，以患者腹部肌肉轻微颤动为度。上巨虚穴，用 1.5 寸毫针直刺 1 寸，小幅度均匀提插捻转 3 次，局部酸胀感为得气；留针期间，每 10 分钟行小幅度均匀提插捻转（3 次）手法一次，共做手法三次。	定位：天枢旁，天枢穴水平旁开 1 寸，脾经和胃经连线中点；腹结旁，腹结穴水平旁开 1 寸，脾经和胃经连线中点；上巨虚旁，上巨虚穴水平旁开，胃经和胆经连线中点。<br>操作方法：仰卧位，皮肤常规消毒。采用 1 寸不锈钢毫针，配以特定长度套管，使针体正好被垂直敲入皮肤 2mm（进入皮下脂肪层），不提插捻转，然后连接电针仪特制电源线电极于双侧天枢旁和腹结旁非穴点针柄上，频率 2-15Hz，电流强度在 0.5ma。特制电源线为中间电线剪断，外表如常；即电针仪显示接通状态，但实际未通电；频次、疗程同试验组。告知患者是一种有效的轻微电流输入，可能感觉不到刺激，但电流是输出的。
疗程	研究周期为 22 周。其中基线期 2 周，治疗期 8 周，随访期 12 周。	研究周期为 22 周。其中基线期 2 周，治疗期 8 周，随访期 12 周。
病例数	40	40
使用仪器	电针仪：韩式穴位神经刺激仪 HANS-200A 型	电针仪：韩式穴位神经刺激仪 HANS-200A 型

生产厂家	华佗牌针灸针：苏州医疗用品厂有限公司<br>电针仪：韩式穴位神经刺激仪 HANS-200A型，南京济生医疗科技有限公司		华佗牌针灸针：苏州医疗用品厂有限公司<br>电针仪：韩式穴位神经刺激仪 HANS-200A 型，南京济生医疗科技有限公司
用法	同上		同上
课题承担单位	中国中医科学院广安门中医院		
本单位临床研究科室	针灸中心	科室负责人	王麟鹏
项目负责人	王麟鹏		
项目参加单位			分中心负责人
1.	首都医科大学附属北京中医医院		王麟鹏
审查文件	1.临床试验方案（20120828 第一版）；2.主要研究者简历；3.伦理申请表；4.知情同意书（20120828 第一版）；5.病例报告表		
试验方法	单盲对照☒	双盲对照☐	非盲法对照☐ 无对照☐
研究者资格	符合要求☒	不符合要求☐	
人员配备及设备条件	符合要求☒	不符合要求☐	
临床研究目的是否适当	适当 ☒	不适当 ☐	
试验条件	符合要求☒	不符合要求☐	
受试者入选方法	合理 ☒	不合理 ☐	
向受试者提供的资料是否完整易懂	是 ☒	否 ☐	
获得知情同意书的方法	适当 ☒	不适当 ☐	
受试者可能遭受的风险和受益是否利大于弊	是 ☒	否 ☐	
受试者因参加临床研究发生不良反应或不良事件时，是否有有效治疗或抢救措施	有 ☒	无 ☐	

委员综合意见：

应到会 9 人	实际到会 6 人	缺席 3 人
伦理委员会委员综合表决意见：		
**意见**	**投票数**	
1. 同意；	6	
2. 做必要的修正后同意；		
3. 不同意；		
4. 终止或暂停已批准的试验。		

其他意见或建议：

1. 如发生严重不良事件及影响研究风险与收益比的非预期不良事件，应及时报告伦理委员会；
2. 如修改课题研究方案和知情同意书、更换主要研究者，应及时通知伦理委员会，进行重新审查，获得批准后再执行。
3. 完成临床研究，请提交试验总结报告表；
4. 自批件生效之日起，每年提前 1 月提交年度/定期跟踪审查申请报告。

综合结论：

依据我国“药物临床试验质量管理规范”、世界医学会“赫尔辛基宣言”制定和确立的人体生物医学研究的伦理和科学标准，经本医学伦理委员会审查，同意按试验研究方案进行“电针治疗严重功能性便秘有效性和安全性国际多中心随机对照试验”。

主任委员签字：

2012 年 5 月 4 日

北京中医医院 医学伦理 委员会

6. Huguosi TCM Hospital of Beijing University of TCM

No. 2012-092 Project		The Efficacy and Safety Study of Electro-acupuncture for Severe Chronic Functional Constipation - a Multicenter, Randomized Controlled Trial	
Sponsor		the 12th National Key Technology Support Program of the Ministry of Science and Technology of the People's Republic of China	
Project No.	2012BAI24B01	PI	Liu Baoyan; Liu Zhishun
		Site PI	Zhou Wei
Participating Centers		Beijing: Guang'anmen Hospital, Beijing TCM Hospital, Huguosi TCM hospital, 301 Hospital, the 3rd Hospital of Zhejiang University of TCM, Dongzhimen Hospita l; Chengdu: West China Hospital of Sichuan University; Hefei: An'hui TCM Hospital; Nanjing: Jiangsu TCM Hospital, Nanjing University of TCM; Guangzhou: Guangdong TCM hospital; Wuhan: Wuhan Hospital of Integrated TCM and Western Medicine; Harbin: Heilongjiang Academy of TCM; Tianjin: the 1st Hospital of Tianjin University of TCM; Shanghai: Shanghai Yueyang Hospital.	

Ethicality	
Comments	Vote
1. Agree	√
2. Agree after the revising	
3. Disagree	

Manning	
Comments	Vote
1. Agree	√
2. Agree after the revising	
3. Disagree	

Protocol	
Comments	Vote
1. Agree	√
2. Agree after the revising	
3. Disagree	

Inform Consent Form

Comments	Vote
1. Agree	√
2. Agree after the revising	
3. Disagree	

Confidentiality declaration	
Comments	Vote
1. Agree	√
2. Agree after the revising	
3. Disagree	

Safety	
Comments	Vote
1. Agree	√
2. Agree after the revising	
3. Disagree	

Conclusion	
Comments	Chair Signature
1. Agree √	Zhang Yunru
2. Agree after the revising	
3. Disagree	

Chief signature: Wang Yi
Committee member signature: Wang Shaohong, Wu You, Zheng Shixie

北京中医药大学附属护国寺中医医院科研课题

伦理委员会综合审查意见表

课题名称	电针治疗严重功能性便秘有效性和安全性多中心随机对照试验		
课题来源	“十二五”国家科技支撑计划		
课题负责人	刘志顺（广安门医院）	分中心负责人	周炜
课题批准号	2012BA124B01		
对课题立题伦理性的审核			
1、同意	√		
2、作必要的修正后同意			
3、不同意			
对研究者、人员配备的审核			
1、同意	√		
2、作必要的修正后同意			
3、不同意			
对临床研究方案的审核			
1、同意	√		
2、作必要的修正后同意			
3、不同意			
对知情同意书的审核			
1、同意	√		
2、作必要的修正后同意			
3、不同意			
对课题保密说明的审核			
1、同意	√		
2、作必要的修正后同意			
3、不同意			
对课题安全性的审核			
1、同意	√		
2、作必要的修正后同意			
3、不同意			
综合审核意见			
1、同意	√		
2、作必要的修正后同意			
3、不同意			
主任签名			
委员签名			

7. 1st Affiliated Hospital of Tianjin University of TCM

No. TYLL2012 【K】004

According to the ministry of health <Biomedical research involving human neighborhood review method (trial) (2007)), the state administration of TCM <TCM clinical research ethical review management regulation> (2010), SFDA's <Drug clinical trial quality management standard"(2003), the WMA < Declaration of Helsinki> (2008), and CIOMS <Human body biomedical research international ethical guidelines> (2002), the trial of < The Efficacy and Safety Study of Electro-acupuncture for Severe Chronic Functional Constipation - a Multicenter, Randomized Controlled Trial > by Dr Fu Lixin and Tianjin TCM Hospital, was approved by our ethical committee.

Please strictly abide by the GCP principle and the protocol approved by this ethical committee (version 1.0 date August 26, 2012), ICF (version 1.0 date August 26, 2012). Clinical registration should be finished before the recruitment. If the situation as followed happened, it should be reported to the ethical committee timely in written report: 1. any change to the protocol and ICF, etc.; 2. changing of PI; 3. serious adverse event; 4. any situation which would affect the trial procedure or increasing the risk to participants; 5. protocol deviation; 6. Termination or pause of the trial.

Our ethical committee will trail the audit of this trial.

Please submit implementation report before October 8, 2013.

Please submit final report after the trail finished.

Validity of this approval: October 8, 2012- October 8, 2015

Ethical Committee of 1st Affiliated Hospital of Tianjin University of TCM

Chief signature: Zhang Jinzhong

Date: October 8, 2012

Contact: Jia Jingyun TEL: 0086-022-27432276

Appendix 1:

Basic information of the submitted Project

Title	The Efficacy and Safety Study of Electro-acupuncture for Severe Chronic Functional Constipation - a Multicenter, Randomized Controlled Trial		
Applicant	1st Affiliated Hospital of Tianjin University of TCM		
Type	Scientific research	Official documents	2012BAI24B01
Discipline	TCM Acupuncture	Site PI	Dr Fu lixin
Participating Centers	Beijing: Guang'anmen Hospital, Beijing TCM Hospital, Huguosi TCM hospital, 301 Hospital, the 3rd Hospital of Zhejiang University of TCM, Dongzhimen Hospita l; Chengdu: West China Hospital of Sichuan University; Hefei: An'hui TCM Hospital; Nanjing: Jiangsu TCM Hospital, Nanjing University of TCM; Guangzhou: Guangdong TCM hospital; Wuhan: Wuhan Hospital of Integrated TCM and Western Medicine; Harbin: Heilongjiang Academy of TCM; Tianjin: the 1st Hospital of Tianjin University of TCM; Shanghai: Shanghai Yueyang Hospital.		

Review type	Preliminary review	Review style	Committee conference
Date	October 8 2012	Address	No 3 meeting room
Document	1. Application for ethical review<br>2. Official document of the trial<br>3. PI CV<br>4. Manning and setting of site<br>5. Investigator handbook<br>6. Protocol (V. August 26 2012)<br>7. Brief summary of prtocol<br>8. ICF (V. August 26 2012)<br>9. CRF		
Blinding	■ YES	☐ NO	
Investigator qualification	■ Qualified	☐ Not Qualified	
Manning	■ Qualified	☐ Not Qualified	
Setting	■ Qualified	☐ Not Qualified	
Participant recruitment	■ Proper	☐ Not Proper	
Document for participant	■ Complete	☐ Not Complete	
ICF	■ Proper	☐ Not Proper	
Paruticipant compensation	■ YES	☐ NO	
Emergency treatment for AE	■ YES	☐ NO	

Contact: Jia Jingyun TEL: 0086-022-27432276

Appendix 2:

Signature of IEC Members;

Date: October 8 2012

Name	title	sex	major	affiliation	signature
Zhang Jinzhong	Chief	Male	Philosophy	Tianjin TCM University	Zhang Jinzhong
Ma Rong	Assistant chief	Male	TCM	1st Hospital of Tianjin University of TCM	Ma Rong
Wu Baoxin	Assistant chief	Male	Western medicine	1st Hospital of Tianjin University of TCM	Wu Baoxin
Yu Tiecheng	Committee member	Male	TCM	1st Hospital of Tianjin	

				University of TCM	
Zhang Junping	Committee member	Male	TCM	1st Hospital of Tianjin University of TCM	Zhang Junping
Wang Shu	Committee member	Male	TCM	1st Hospital of Tianjin University of TCM	Wang Shu
Hu Siyuan	Committee member	Male	TCM	1st Hospital of Tianjin University of TCM	Hu Siyuan
Guo Congrong	Committee member	Female	TCM	1st Hospital of Tianjin University of TCM	Guo Congrong
Li Jin	Committee member	Male	TCM Pharmacy	Shitian Pharmacy company	Li Jin
Cao Lihua	Committee member	Female	Law	Changshi Law firm	Cao Lihua
Cao Suchun	Committee member	Female	Policy management	Community	Cao Suchun
Liu Xinqiao	Committee member	Male	TCM	1st Hospital of Tianjin University of TCM	
Zhang Chongquan	Committee member	Male	Law	Changshi Law firm	

天津中医药大学第一附属医院医学伦理委员会
IEC of The First Affiliated Hospital of Tianjin University of Traditional Chinese Medicine

审　查　批　件

Approval Notice

伦理批件号：TYLL2012[K]字 004

根据卫生部《涉及人的生物医学研究伦理审查办法》（2007）、国家中医药管理局《中医药临床研究伦理审查管理规范》（2010）、国家食品药品监督管理局《药物临床试验伦理审查工作指导原则》（2010）、《药物临床试验质量管理规范》（2003），以及世界医学会《赫尔辛基宣言》（2008）、国际医学科学组织理事会《人体生物医学研究国际伦理指南》（2002）的伦理原则，经天津中医药大学第一附属医院医学伦理委员会 2012 年 10 月 8 日第六次会议审查，同意由申办者天津中医药大学第一附属医院和主要研究者傅立新共同申请的电针治疗严重功能性便秘有效性和安全性国际多中心随机对照试验临床研究项目开展临床研究工作。

请申办者、研究人员严格遵循 GCP 规定和本伦理委员会批准的方案（版本号：VERSION 1.0 版本日期：20120826）、知情同意书（版本号：VERSION 1.0 版本日期：20120826）开展临床研究。在研究开始前，须完成临床试验注册。该项目进行中如发生下列情况，须及时书面报告本伦理委员会：①对临床方案、知情同意书等的任何修改；②更换主要研究者；③发生严重不良事件；④出现任何可能影响试验进行或增加受试者危险的情况；⑤出现违反方案情况；⑥暂停或提前终止临床研究。

本伦理委员会将对该项目跟踪审查。

请于 2013 年 10 月 8 日前 1 个月提交研究进展报告。

该项目完成后，请向本伦理委员会提交结题报告。

本批件有效期为 2012 年 10 月 8 日至 2015 年 10 月 8 日。

天津中医药大学第一附属医院医学伦理委员会

主任委员签字：张金瑄

日　　期：2012.10.8.

联系人：贾晨蕴　　联系电话：022-27432276

附件 1

天津中医药大学第一附属医院医学伦理委员会

IEC of The First Affiliated Hospital of Tianjin University of Traditional Chinese Medicine

提交审查项目基本情况

Basic Information of The Submitted Project

项目名称	电针治疗严重功能性便秘有效性和安全性国际多中心随机对照试验临床研究		
申办单位	天津中医药大学第一附属医院		
临床研究分类	科研课题	临床研究批文	课题编号 **2012BAI24B01**
所属专业	中医针灸专业	主要研究者	傅立新
研究单位	天津中医药大学第一附属医院、中国中医科学院广安门医院、北京中医药大学东直门医院、北京中医医院、北京市护国寺中医院、301 医院、四川大学华西医院、南京中医药大学、江苏省中医院、广东省中医院、安徽省中医院、浙江中医药大学附属第三医院、上海中医药大学附属岳阳中西医结合医院、武汉中西医结合医院、黑龙江省中医研究院		
审查类型	初始审查	审查方式	会议审查
审查时间	**2012** 年 **10** 月 **8** 日	审查地点	行政楼第三会议室
审阅资料	1、提请伦理审查申请书<br>2、临床研究批文<br>3、主要研究者履历<br>4、专业科室人员配备及设备设施情况介绍<br>5、研究者手册<br>6、临床试验方案（版本号：VERSION 1.0 版本日期：20120826）<br>7、临床试验方案摘要<br>8、知情同意书（版本号：VERSION 1.0 版本日期：20120826）<br>9、CRF		

试验是否采用盲法？	是 √	否	（请在相应处打 √）
研究者资格是否符合要求？	符合 √	不符合	（请在相应处打 √）
人员配备是否符合要求？	符合 √	不符合	（请在相应处打 √）
设备是否符合要求？	符合 √	不符合	（请在相应处打 √）
受试者招聘方法是否恰当？	恰当 √	不恰当	（请在相应处打 √）
向受试者提供的资料是否完整？	完整 √	不完整	（请在相应处打 √）
《知情同意书》是否规范？	规范 √	不规范	（请在相应处打 √）
受试者参加试验有无补偿？	有	无 √	（请在相应处打 √）
发生不良反应或意外时	有及时抢救措施 √	无及时抢救措施	（请在相应处打 √）

联系人：贾景蕴　　联系电话：022-27432276

附件 2

天津中医药大学第一附属医院医学伦理委员会
IEC of The First Affiliated Hospital of Tianjin University of Traditional Chinese Medicine

与会伦理委员会成员签到表

Signature of IEC Members

会议日期：2012 年 10 月 8 日

姓　名	职　务	性别	专业情况	单　位	签　字
张金钟	主任委员	男	哲　学	天津中医药大学	
马　融	副主任委员	男	中医学	天津中医药大学第一附属医院	
吴宝新	副主任委员	男	医　学	天津中医药大学第一附属医院	
于铁成	委员	男	中医学	天津中医药大学第一附属医院	
张军平	委员	男	中医学	天津中医药大学第一附属医院	
王　舒	委员	男	中医学	天津中医药大学第一附属医院	
胡思源	委员	男	中医学	天津中医药大学第一附属医院	
郭从容	委员	女	中医学	天津中医药大学第一附属医院	
李　进	委员	男	中药学	天津市石天药业责任有限公司	
曹丽华	委员	女	法　律	长实律师事务所	
曹素纯	委员	女	政　工	社区	
刘新桥	委员	男	中医学	天津中医药大学第一附属医院	
张崇泉	委员	男	法　律	长实律师事务所	

8. Dongzhimen Hospital of Beijing University of TCM

Approval notice Template

Review No. ESCL-BDY-2012-52 Approval No: ESCL-BDY-2012-52

Title: The Efficacy and Safety Study of Electro-acupuncture for Severe Chronic Functional Constipation - a Multicenter, Randomized Controlled Trial

Applicant: Dongzhimen Hospital of Beijing University of TCM

Research type: the 12th National Key Technology Support Program of the Ministry of Science and Technology of the People's Republic of China (2012BAI24B01)

Department: Acupuncture department Site PI: Dr Zhao Jiping

Ethical Review: ☐ Conference review ■ fast review

Member: anticipated 3 persons

According to SFDA's <TCM clinical research ethical review management regulation> (2010) <Drug clinical trial quality management standard"(2003), <TCM herb protect guidance> (2008), the ministry of health <Biomedical research involving human neighborhood review method (trial) (2007)), the state administration of TCM <TCM clinical research ethical review management regulation> (2010), and CIOMS <Human body biomedical research international ethical guidelines> (2002), the trial of < The Efficacy and Safety Study of Electro-acupuncture for Severe Chronic Functional Constipation - a Multicenter, Randomized Controlled Trial > by Dr Fu Lixin and Tianjin TCM Hospital, was:	■ Approved<br>☐ Not Approved<br>☐ Terminated<br>☐ Paused

Comment:

Approved

Note:

The validity of this approval is 1 year. Site PI must abide by the approved documents. If the trial could not accomplished before the validity (including the statistical analysis), please submit for review 1 month before the deadline. If the trial accomplished in the validity, please submit the final report. If there is any adverse event related to the trial occurred, please reported to the committee. If there is any change about the protocol, ICF or investigators, modification application must be submitted to the committee and get approved.

Chief Date: October 23, 2012

Assistant chief ■ Address: Dongzhimen Hospital of Beijing University of TCM

Trailing frequency: ☐ 3 months ☐ 6 months ■ 12months

Contact: Shang Jianwei 0086-010-84013229

Committee list and signature

Name	sex	title	major	signature
Liu Hongfang	Female	Assistant chief	endocrinology	Liu Hongfang
Yang Bohua	Male	Committee member	Surrounding blood-vessel	Yang Bohua
Wang Pengwen	Female	Committee member	pharmacology	Wang Pengwen

北京中医药大学东直门医院医学伦理委员会

IRB of Dongzhimen Hospital affiliated to Beijing University of Chinese Medicine

伦理审查批件

Approval Notice Template

受理序号：ECSL-BDY-2012 -52　　批件号：ECPJ-BDY-2012 -52

项目名称：电针治疗严重功能性便秘有效性和安全性国际多中心随机对照试验

申办单位：东直门医院

项目类别："十二五"国家科技支撑课题　　课题编号：2012BAI24B01

承担科室：针灸科　　主要研究者：赵吉平

伦理审查方式：□会议审查　　☑快速审查

应到会　人，出席本次会议人员 3 人，回避　人，缺席　人。

根据中华人民共和国国家食品药品监督管理局（SFDA）《药物临床试验伦理审查工作指导原则》（2010 年）、《药物临床试验质量管理规范》（2003）、《中药品种保护指导原则》（2009），世界医学会《赫尔辛基宣言》（2008），卫生部《涉及人的生物医学研究伦理审查办法》（2007），国家中医药管理局《中医药临床研究伦理审查管理规范》（2010）以及国际医学科学组织委员会《人体生物医学研究国际道德指南》（2002）的伦理原则，经本伦理委员会审查决定：	☑同意临床研究方案<br>□不同意临床研究方案<br>□终止临床研究方案<br>□暂停临床研究方案

审查意见：

同意本项目应用。

注：**本批件自签发日期有效期一年**，研究负责人必须严格使用经审查同意的知情同意书文本和研究方案。如伦理审查批件失效时不能完成所有的临床研究（包括统计分析），请在本批件失效前一个月，递交持续审查申请。如研究结束并在审查有效期内，请递交研究结题报告。研究中发生涉及受试者或其他人风险的任何预期或非预期的不良事件，应立刻报告本伦理委员会；任何研究方案、知情同意书的修改包括研究人员得变更，必须递交研究方案修改申请表，经伦理委员会审查获得批准后执行。

主任委员□副主任委员□签字：　　时 间：2012年10月23日

北京中医药大学东直门医院医学伦理委员会　　地 点：东直门医院

本项目持续审查频率□3个月 □6个月 ☑12个月　联系人：商建伟　(010) 84013229

北京中医药大学东直门医院医学伦理委员会

出席本次审查项目委员名单

项目名称：电针治疗严重功能性便秘有效性和安全性国际多中心随机对照试验

成员	性别	伦理委员会职务	专业	签名
柳红芳	女	副主任	肾病内分泌	
杨博华	男	委员	周围血管	
王蓬文	女	委员	药理学	

9. 3rd Affiliated Hospital of Zhejiang University of TCM

Trial name	The Efficacy and Safety Study of Electro-acupuncture for Severe Chronic Functional Constipation - a Multicenter, Randomized Controlled Trial				
Research rang	Sep 1 2012- Sep 1 2015		Type	Clinical trial	
Total sample size	2100	Sample size for site	80	Other sites	2020
Research objective	To evaluate the effect and safety of electro-acupuncture for Severe Chronic Functional Constipation				
Supervisor	Ministry of Science and Technology		Project No	2012BAI24B00	
Applicant	3rd Affiliated Hospital of Zhejiang University of TCM		Site Chief	Dr Fang Jianqiao	
Site	3rd Affiliated Hospital of Zhejiang University of TCM		Department	Acupuncture departement	
Site PI	Dr Fang Jianqiao		Title	Professor	
Approval No	ASLL-KY-2012-002		Validity	Sep 2012- Dec 2015	
Document			Version	Date	
√	Application for ethical review		1.0	January 15. 2012	
√	Clinical protocol		4.0	January 15. 2012	
√	ICF		2.0	January 15. 2012	
√	Investigator Handbook		3.0	January 15. 2012	
√	CRF		2.0	January 15. 2012	

Comment:

The design of this trial is scientific for human body research.

The trial is approved by our ethical committee.

3rd Affiliated Hospital of Zhejiang University of TCM

Ethical Committee

August 20 2012

Contact: 0086-0571-88393504

Address: No 219, Moganshan road, Hangzhou, Zhejiang, China

浙江中醫藥大學附屬第三醫院

临床试验伦理委员会审批件

试验项目名称	针灸疗效国际多中心临床评价研究<br>——电针治疗严重功能性便秘有效性和安全性评价				
研究期限	2012-09-01 至 2015-12-31			试验类别	临床试验
受试者总例数	2100	申请医疗机构承担	80	其他机构承担	2020
试验目的	评价电针治疗严重功能性便秘的有效性和安全性				
监督管理部门	中华人民共和国科学技术部			项目编号	2012BAI24B00
申办单位	浙江中医药大学附属第三医院			主要负责人	方剑乔
医疗机构	浙江中医药大学附属第三医院			临床研究部门	针灸科
项目负责人	方剑乔			职称	教授
批件文号	ZSLL-KY-2012-002			批件有效期	2012.9-2015.12

	审查文件名称	版本	日期
√	伦理审查申请书	1.0	2012.1.15
√	临床研究方案	4.0	2012.1.15
√	知情同意书样本	2.0	2012.1.15
√	研究者手册	3.0	2012.1.15
√	病例报告表	2.0	2012.1.15

审批意见：

该项目设计科学，研究方法符合人体临床试验伦理学要求，同意进行临床试验研究。

浙江中医药大学附属第三医院
伦理委员会（盖章）
2012 年 08 月 20 日

伦理委员会联系电话：0571-88393504
联系地址：浙江省杭州市莫干山路 219 号 （310005）

10. 301 Hospital

No. 2012-037

<table>
<tr><td rowspan="7">Project</td><td>Project title</td><td colspan="3">The Efficacy and Safety Study of Electro-acupuncture for Severe Chronic Functional Constipation - a Multicenter, Randomized Controlled Trial</td></tr>
<tr><td>Resource</td><td colspan="3">International Collaboration ☐
National project ■
Army project ☐
Beijing project ☐
Hospital project ☐
Others ☐</td></tr>
<tr><td>Investigator Attended</td><td colspan="3">Guan Ling, Xia Hongqing, Wang Lili, Jiang Yuebo, Yang Yiling</td></tr>
<tr><td>Project No</td><td>2012BAI24B01</td><td>Research range</td><td>Oct 8.2012- Sep 30, 2014</td></tr>
<tr><td>Department</td><td>Acupuncture department</td><td>Site PI</td><td>Guan Ling</td></tr>
<tr><td>Title</td><td>Assistant Chief Physician</td><td>Contact</td><td>1350575830;66937410</td></tr>
<tr><td colspan="4"></td></tr>
<tr><td>Docmument</td><td colspan="3">1. Protocol (Version 1.0/20120917)
2. ICF
3. CRF (Version 1.0/20120916)
4. Research medical record</td></tr>
<tr><td rowspan="2">Comment</td><td>Attended</td><td>13 persons</td><td>Vote</td><td>13</td><td>Avoid</td><td>0</td></tr>
<tr><td colspan="6">Agree 13
Not agree 0
Agree after revision 0
Re-review after revision 0
Terminated or pause 0</td></tr>
<tr><td>Conclusion</td><td colspan="6">■ Agree 13
☐ Not agree 0
☐ Agree after revision 0
☐ Re-review after revision 0
☐ Terminated or pause 0
The ethical committee of 301 Hospital approved the trial as Agree after revision on October 23, 2012. The ethical committee of 301 Hospital approved the trial as Agree on November 21, 2012.
Final decision: Agree.

Did this trial accept the continuing review during the process? ■ Yes ☐ No
The frequency for trailing is ☐ 6 months ■ 12 months</td></tr>
<tr><td colspan="7">Signature of chief:
STAMP</td></tr>
</table>

Contact: No 28, Fuxing Road, Haidian District, Beijing, China 100853 0086-010-66937166

中国人民解放军总医院医学伦理委员会
临床科研课题审批件

【2012】伦审科研第（037）号

评审项目	项目名称	电针治疗严重功能性便秘有效性和安全性国际多中心随机对照试验		
	课题来源	国际合作课题 □ 国家级科研课题■ 军队科研课题 □<br>北京市科研课题 □ 医院科研课题 □ 其它 □		
	参加研究人员	关 玲 夏鸿清 王莉莉 姜岳波 杨一玲		
	课题编号	2012BAI24B01	起始时间	2012-10-8~2014-9-30
	科 室	针灸科	课题负责人	关 玲
	职 称	副主任医师	联系电话	13520575830；66937410
受理审查文件	□ 1.临床研究方案（VERSION1.0/20120917）<br>□ 2.知情同意书（注明版本号/版本日期）<br>□ 3.病例报告表（VERSION1.0/20120916）<br>□ 4.研究病历（注明版本号/版本日期）			
委员意见	出席	13 人	投票	13 人	回避	0 人
	同意 13 人 不同意 0 人 作必要的修正后同意 0 人<br>作必要修改后重审 0 人 终止或暂停已批准的试验 0 人			
结论	■同意 □不同意 □作必要的修正后同意 □作必要修改后重审 □终止或暂停已批准的试验<br>解放军总医院伦理委员会于 2012 年 10 月 23 日会议审查了该项目的方案及知情同意书等研究资料。审查结果：作必要修改后重审。解放军总医院伦理委员会于 2012 年 11 月 21 日会议审查了该项目的修改资料。审查结果：同意。<br>该研究进行过程中将接受伦理委员会的持续审查？■是 □否<br>审查频度为研究批准之日起 □6 个月 ■12 个月			
	主任委员/授权者签名：<br>医学伦理委员会（盖章）：<br>2012 年 12 月 3 日			

地址：北京市海淀区复兴路 28 号 邮编：100853 电话：010-66937166

11. Wuhan Hospital of Integrated TCM and Western Medicine
No.2012-5

Project	The Efficacy and Safety Study of Electro-acupuncture for Severe Chronic Functional Constipation - a Multicenter, Randomized Controlled Trial		
Applicant	Wuhan Hospital of Integrated TCM and Western Medicine Acupuncture department		
Site PI	Zhang Hongxing	Ethical Approval	2012-5
Ethical Committee	Ethical Committee of Wuhan Hospital of Integrated TCM and Western Medicine		
Document:<br>1. Application form for ethical review<br>2. Feasibility, PI CV, manning<br>3. ICF<br>4. Protocol			
Comment:<br>1. The trial of < The Efficacy and Safety Study of Electro-acupuncture for Severe Chronic Functional Constipation - a Multicenter, Randomized Controlled Trial > is approved by our committee.<br>2. During the process, please follow the relevant laws, regulations and rules such as <Declaration of Helsinki> , etc.<br>3. Annual report should be submitted to the committee. Any change of the trial should be reported to the committee and get approved.<br><br>Chief signature: Zhang Wenjun<br>Date : August 21, 2012<br><br>This ethical committee is responsible to the ethical and moral issue in approved trials.			

Contact: No 215, Zhonshan Road, Wuhan, Hubei, China, 430022 TEL:027-85332012

武汉市第一医院
医学伦理委员会伦理审查批复件

武卫一院伦审〔2012〕5号

项目名称	电针治疗严重功能性便秘有效性和安全性国际多中心随机对照试验		
申报单位	武汉市第一医院针灸科		
项目负责人	张红星	伦理审批号	〔2012〕5号
审批伦理委员	武汉市第一医院医学伦理委员会		

伦理委员会收到以下相关文件：

1、医学伦理审查的申请

2、项目的可行性分析，研究者的资格、经验、人员组成和履历

3、知情同意书的样本

4、研究方案

医学伦理委员会意见：

1、经本伦理委员会审查：同意我院针灸科参与北京广安门医院进行“电针治疗严重功能性便秘有效性和安全性国际多中心随机对照试验”课题的研究。

2、在项目开展过程中，应遵守国际《赫尔辛基宣言》及我国的伦理原则、道德标准及相关法律、法规、制度等。

3、每年向医学伦理委员会报告一次工作情况。如对病种、实施方案或知情同意书等进行任何修改，均应及时向医学伦理委员会书面报告，经同意后方可继续进行。

主任委员：

批复日期：二〇一二年八月二十一日

武汉市第一医院 医学伦理委员会

声明：本委员会仅对备案的临床研究项目中涉及的伦理与道德问题负责

联系地址：武汉市中山大道215号，武汉市第一医院医学伦理委员会　邮编：430022
联系电话：027—85332012

12. Yueyang Hospital of Shanghai University of TCM

Approval Notice template

No. 2013-006

Project	The Efficacy and Safety Study of Electro-acupuncture for Severe Chronic Functional Constipation - a Multicenter, Randomized Controlled Trial	Project official no	
Type	RCT	Duration	1year
Site name	Yueyang Hospital of Shanghai University of TCM	Site PI	Dong Guirong
Committee member	Jin Liguo, Xu Lingling, Chen Yunfei, Chang Shixin, Fan Minsheng, Zhou Zheng, Ren Li		
Address	No 110, Ganhe Road, Hongkou District, Shanghai, China		
Date	Feb 28, 2013		
Comment	According to the SFDA's <Drug clinical trial quality management standard"(2003), the WMA < Declaration of Helsinki> (2008), and CIOMS <Human body biomedical research international ethical guidelines> (2002), the committee discussed the documents:<br>Application form for ethical review<br>Protocol (20130218)<br>ICF (20130315)<br>PI CV; Investigator list<br>CRF (1.0_20120916), patient diary<br>Research handbook<br>PI Hospital ethical review approval notice<br>The trial of < The Efficacy and Safety Study of Electro-acupuncture for Severe Chronic Functional Constipation - a Multicenter, Randomized Controlled Trial > by Dr Fu Lixin and Tianjin TCM Hospital, was approved by our ethical committee.<br>Please contact us if you have any different comment about the feasibility.<br>During the experiment, if there is any change about the PI, the clinical study scheme, ICF, etc., the applicant should submit the revised protocol for re-review. If there is any serious adverse events occurs, the applicant should submit the serious adverse event reports; After emergency report, details of the follow-up report should be reported in serious adverse events report in 24 hours.		
Chief signature	Jin Liguo		
Note	The validity of this approval is 12 month. If the trial could not be accomplished, please submit trailing audit application.<br>Contact: Xiao Xiayi TEL: 65161782-2419		

Yueyang Hospital of Shanghai University of TCM

Ethical Committee STAMP

March 18, 2013

Sign-in Sheet of full board Meeting
Date: Feb 28, 2013
Project: The Efficacy and Safety Study of Electro-acupuncture for Severe Chronic Functional Constipation - a Multicenter, Randomized Controlled Trial
Signature:

Name	Sex	Major	Signature
Jin Liguo	Male	Medicine management	Jin Liguo
Xu Lingling	Femal	Pharmacology	Xu Lingling
Chen Yunfei	Male	Acupuncture	Chen Yunfei
Chang Shixin	Male	Imageology	Chang Shixin
Fan Minsheng	Male	Ethics	Fan Minsheng
Zhou Zheng	Male	Law	Zhou Zheng
Ren Li	Male	Law	Ren Li

上海中医药大学附属岳阳中西医结合医院伦理委员会

IRB of Yueyang Hospital of Integrated Traditional Chinese and Western Medicine, Shanghai University of TCM

伦理审查批件

Approval Notice Template

伦理审议批件号：上海中医药大学附属岳阳中西医结合医院伦理委员会 2013 伦理审查 006 号

研究名称	电针治疗严重功能性便秘有效性和安全性多中心随机对照试验		临床试验批准文号	
研究类型	病例对照	研究周期	一年	
研究单位和研究者	上海中医药大学附属岳阳中西医结合医院　东贵荣			
伦理委员会审议成员	金利国、徐玲玲、陈云飞、常时新、樊民胜、周正、任力			
伦理委员会地址	上海市虹口区甘河路 110 号			
审议时间	2013 年 2 月 28 日			
审议结论	根据中华人民共和国国家药品监督管理局 2003 年颁布实施的《药物临床试验质量管理规范》以及《赫尔辛基宣言》和国际医学科学组织委员会颁布的《人体生物医学研究国际道德指南》的道德原则。本伦理委员会的全体成员审阅并讨论了下列有关材料：<br>1. 伦理审查申请表<br>2. 研究方案（20130218）<br>3. 知情同意书（20130315）<br>4. 主要研究者简历、参加研究人员名单<br>5. 病例观察表（1.0_20120916）、患者日记<br>6. 工作手册<br>7. 组长单位伦理批件<br>本伦理委员会经表决同意你们自即日起开展“电针治疗严重功能性便秘有效性和安全性多中心随机对照试验”；<br>并要求：上述资料未经本委员会批准，不得作任何修改；试验过程中如发生严重不良事件，应立即（24 小时内）报告本委员会；如临床试验方案、知情同意书及研究者有任何更改，应及时通知伦理委员会，得到重新批准。			
主任委员签字				
备注	该批件有效期一年，自批件生效日起 12 月内未完成研究的，请向伦理委员会提交跟踪审查申请。<br>联系人：肖夏懿 电话：65161782*2419			

上海中医药大学附属岳阳中西医结合医院

医学伦理委员会（盖章）

2013 年 3 月 18 日

上海中医药大学附属岳阳中西医结合医院伦理委员会

IRB of Yueyang Hospital of Integrated Traditional Chinese and Western Medicine, Shanghai University of TCM

会议签到表

Sign-in Sheet of Full Board Meeting

会议日期：2013 年 2 月 28 日

审查项目：电针治疗严重功能性便秘有效性和安全性多中心随机对照试验

伦理委员会到会委员签名：

姓名	性别	专业情况	签名
金利国	男	医学、管理	
徐玲玲	女	药学	
陈云飞	男	针灸学	
常时新	男	影像学	
樊民胜	男	社会科学、伦理学	
周　正	男	法律	
任　力	男	法律	

13. Nanjing University of TCM

No. KY2012008

Project	The Efficacy and Safety Study of Electro-acupuncture for Severe Chronic Functional Constipation - a Multicenter, Randomized Controlled Trial	
No.	the 12th National Key Technology Support Program of the Ministry of Science and Technology of the People's Republic of China (2012BAI24B01)	
Site PI	Zhang Jianbin; Chen Chaoming; acupuncture department	
Sponsor		
PI Hospital	Guang An Men Hospital; Nanjing University of TCM; Nanjing Hospital of TCM	
Review type	Conference review	
Committee member	Gao Hong, Yu Heming, Wu Suling, Zhang Zhongai, Shen Hong, Niu Lihong, Lei Maohua, Zheng Xueping, Chen Yan	
Document	1. Ethics requirement	■ Qualified ☐ Not Qualified
	2. PI and Scientific requirement	■ Qualified ☐ Not Qualified
	3. Subjects rights and interests guarantee	■ Qualified ☐ Not Qualified
	4. Risk control and ethical management	■ Qualified ☐ Not Qualified
	5. Fairness and justice	■ Qualified ☐ Not Qualified
	6. Effect evaluation	■ Qualified ☐ Not Qualified
Conclusion	1. According to the the ministry of health <Biomedical research involving human neighborhood review method (trial) (2007)), WMA < Declaration of Helsinki> (2008), CIOMS <Human body biomedical research international ethical guidelines> (2002),The trial of < The Efficacy and Safety Study of Electro-acupuncture for Severe Chronic Functional Constipation - a Multicenter, Randomized Controlled Trial > by Dr Fu Lixin and Tianjin TCM Hospital, was approved by our ethical committee.<br>2. If there is any serious adverse events occurs, the applicant should submit the serious adverse event reports; After emergency report, details of the follow-up report should be reported.<br>3. During the experiment, if there is any change about the PI, the clinical study scheme, ICF, etc., the applicant should submit the revised protocol for re-review.	
Chief Signature	Agree	
STAMP		
Address	No 7 meeting room, DATE: July 9 2012	
Contact	Zhao Xuelong, 02552276305; 15951896765	

南京市中医院伦理委员会

涉及人的生物医学研究/医疗新技术伦理审查报告

南京市中医院伦理委员会 KY2012008

项目名称	电针治疗严重功能性便秘有效性和安全性国际多中心随机对照试验		
项目编号	“十二五”国家科技支撑计划项目（项目编号：2012BAI24B01）		
项目负责人	张建斌 陈朝明	所在科室	针灸科
组织实施单位			
研究单位	中国中医科学院广安门医院、南京中医药大学、南京市中医院		
审查方式	会议审查		
审查委员	高虹、虞鹤鸣、吴素玲、张钟爱、沈红、钮立红、雷茂华、郑雪平、陈彦		
审查材料	项目申请书		
审查内容	1. 伦理规范要求 ✓符合 □ 不符合<br>2. 研究者及科学性要求 ✓ 符合 □ 不符合<br>3. 受试者权益保障 ✓ 符合 □ 不符合<br>4. 风险控制及伦理处理措施 ✓ 符合 □ 不符合<br>5. 公平公正原则要求 ✓ 符合 □ 不符合<br>6. 技术效果评价 ✓ 符合 □ 不符合		
审查结论	1. 根据卫生部《涉及人的生物医学研究伦理审查办法（试行）》以及《赫尔辛基宣言》和国际医学科学组织委员会颁布的《人体生物医学研究国际道德指南》的道德原则，经本伦理委员会审查决定，同意电针治疗严重功能性便秘有效性和安全性国际多中心随机对照试验开展。<br>2. 如发生严重不良事件，应及时报告伦理委员会。<br>3. 如方案、知情同意书的任何修改，主要研究者更换，应及时通知伦理委员会，重新审查，获得批准后执行。		
主任委员签字			
伦理委员会签章			
审查地点	门诊七楼会议室	审查时间	2012-07-09
联系热线	赵学龙，02552276305（0）；15951896765		

14. Jiangsu Hospital of TCM

Approval No	2012NL-052-02		
Project	The Efficacy and Safety Study of Electro-acupuncture for Severe Chronic Functional Constipation - a Multicenter, Randomized Controlled Trial		
Resource	the 12th National Key Technology Support Program of the Ministry of Science and Technology of the People's Republic of China (2012BAI24B01)		
Applicant	Jiangsu Hospital of TCM; Guang An Men Hospital		
Site PI	Sun Jianhua		
Type	Re-review	Style	Fast review
Date	January 7, 2013	Address	
Committee member	Xue Mingxin		
Document	Re-review application<br>Protocol (revised) VERSION 1.1 DATE 2012-12-25<br>ICF (revised) VERSION 1.1 DATE 2012-12-25<br>Recruitment document (revised)		
Comment	According to the ministry of health <Biomedical research involving human neighborhood review method (trial) (2007)), the SFDA's <Drug clinical trial quality management standard"(2003), <medical device clinical research regulation>(2004), WMA < Declaration of Helsinki> and CIOMS <Human body biomedical research international ethical guidelines>, the trial of < The Efficacy and Safety Study of Electro-acupuncture for Severe Chronic Functional Constipation - a Multicenter, Randomized Controlled Trial > was approved by our ethical committee.<br>Please follow the GCP principles and the approved protocol by ethical committee, protect the right of subject. The clinical registration should be finished before the trial started.<br>During the experiment, if there is any change about the PI, the clinical study scheme, ICF, etc., the applicant should submit the revised protocol for re-review.<br>If there is any serious adverse events occurs, the applicant should submit the serious adverse event reports; After emergency report, details of the follow-up report should be reported in serious adverse events report as soon as possible.<br>The summary report need to be submitted after completing the clinical trial 1 month before the deadline of this approval.<br>Please submit the annual inspection and regular tracking report. When any situation which may significantly impact tests or increase the risk of the subjects, the applicant should submit a written report to the ethics committee timely.<br>If there is any participant who did not qualified for the inclusion criteria		

	or qualified for the exclusion, giving wrong therapy or dose, giving solutions such as follow on the prohibited drug combination of situation or any other situation which may affect the rights and interests of subjects, thus the applicant/monitor/investigator should submit the report.
Trailing audit	Report should be submitted before January 7, 2014
Validity	12 months
Contact	Wu Jing 31618
Chief Signature	Liu Shende
Ethical Committee	Ethical Committee of Jiangsu Hospital of TCM
Date	January 7, 2013

伦理审查批件

批件号	2012NL-052-02		
项目名称	电针治疗严重功能性便秘有效性和安全性多中心随机对照试验		
项目来源	“十二五”国家科技支撑计划		
研究单位	江苏省中医院，中国中医科学院广安门医院		
主要研究者	孙建华		
审查类别	复审申请	审查方式	快速审查
审查日期	2013 年 01 月 07 日	审查地点	
审查委员	薛明新		
审查文件	复审申请<br>修改的临床研究方案 版本号：VERSION1.1 版本日期：2012-12-25<br>修改的知情同意书 版本号：VERSION1.1 版本日期：2012-12-25<br>修改的招募材料		

审查意见

根据卫生部《涉及人的生物医学研究伦理审查办法（试行）》(2007)、SFDA《药物临床试验质量管理规范（2003）》、《医疗器械临床试验规定（2004）》、WMA《赫尔辛基宣言》和CIOMS《人体生物医学研究国际道德指南》的伦理原则，经本伦理委员会审查，同意按所批准的临床研究方案、知情同意书、招募材料开展本项研究。

请遵循 GCP 原则、遵循伦理委员会批准的方案开展临床研究，保护受试者的健康与权利。研究开始前，请申请人完成临床试验注册。研究过程中若变更主要研究者，对临床研究方案、知情同意书、招募材料等的任何修改，请申请人提交修正案审查申请。发生严重不良事件，请申请人及时提交严重不良事件报告；紧急报告之后，尽快提交详细的严重不良事件随访报告。请按照伦理委员会规定的年度/定期跟踪审查频率，申请人在截止日期前 1 个月提交研究进展报告；申办者应当向组长单位伦理委员会提交各中心研究进展的汇总报告；当出现任何可能显著影响试验进行、或增加受试者危险的情况时，请申请人及时向伦理委员会提交书面报告。研究纳入了不符合纳入标准或符合排除标准的受试者，符合中止试验规定而未让受试者退出研究，给予错误治疗或剂量，给予方案禁止的合并用药等没有遵从方案开展研究的情况；或可能对受试者的权益/健康、以及研究的科学性造成不良影响等违背 GCP 原则的情况，请申办者/监查员/研究者提交违背方案报告。申请人暂停或提前终止临床研究，请及时提交暂停/终止研究报告。完成临床研究，请申请人提交结题报告。本项临床试验应当在批准之日起一年内实施，逾期未实施的，本批件自行废止。

年度/定期跟踪审查频率	请于 2014 年 01 月 07 日前 1 个月提交研究进展报告
有效期	12 个月
联系人与联系电话	吴静　31618
主任委员签字	
伦理委员会	南京中医药大学附属医院（江苏省中医院）伦理委员会（盖章）
日期	2013 年 01 月 07 日

15. Anhui Hospital of TCM

Approval No	2012KSZL01
Project	The Efficacy and Safety Study of Electro-acupuncture for Severe Chronic Functional Constipation - a Multicenter, Randomized Controlled Trial
Resource	the 12th National Key Technology Support Program of the Ministry of Science and Technology of the People's Republic of China (2012BAI24B01)
PI Hospital	Guang An Men Hospital
Site Hospital	Anhui Hospital of TCM
Site PI	Qin Lihong
Type	Fast review
Document	1. Application for ethical review<br>2. Protocol (V.1.0 20120917)<br>3. ICF (V.1.0 20120917)<br>4. Recruitment document<br>5. CRF<br>6. Investigator handbook<br>7. PI CV; Investigator list<br>8. PI hospital ethical approval<br>9. Technical cooperation contract
Comment	According to the SFDA's <Drug clinical trial quality management standard"(2003), <Ethical review regulation for drug> (2010), the ministry of health <Biomedical research involving human neighborhood review method (trial) (2007), the state administration of TCM < Ethical review regulation for TCM> (2010), WMA < Declaration of Helsinki> the trial of < The Efficacy and Safety Study of Electro-acupuncture for Severe Chronic Functional Constipation - a Multicenter, Randomized Controlled Trial > was approved by our ethical committee.
Ethical Committee Declaration	If there is any serious adverse events occurs, the applicant should submit the serious adverse event reports; After emergency report, details of the follow-up report should be reported in serious adverse events report as soon as possible. During the experiment, if there is any change about the PI, the clinical study scheme, ICF, etc., the applicant should submit the revised protocol for re-review.<br>Please submit the annual inspection and regular tracking report.<br>The summary report need to be submitted after completing the clinical trial before the deadline of this approval.
Validity	24 months Trialing audit date: 2013-09-20
Contact	Xu Shuqin tel:0551-62838532
Chief Signature	Run Yun 2012-09-18
Ethical Committee of Anhui Hospital of TCM<br>STAMP	

安徽中医学院第一附属医院伦理委员会

伦理审查批件

伦理审查批件号	2012KSZL01		
项目名称	电针治疗严重功能性便秘有效性和安全性多中心随机对照试验		
自助来源	国家十二五科技支撑计划		
组长单位	中国中医科学院广安门医院		
研究单位	安徽省中医院		
主要研究者	秦黎虹		
审查类别/<br>审查方式	初始/快速审查		
批准文件	伦理审查申请表<br>临床研究方案（版本号/版本日期：VERSION1.0_20120917/2012 年 9 月）<br>知情同意书（版本号/版本日期：VERSION1.0_20120917/2012 年 9 月）<br>招募受试者的材料<br>病例报告表<br>研究者手册<br>主要研究者专业履历及研究人员名单、职责分工<br>组长单位伦理审查批件<br>技术合作合同		
审<br>查<br>意<br>见	根据我国食品药品监督管理局“药物临床试验质量管理规范”(2003 年)，“药物临床试验伦理审查工作指导原则”(2010 年)，卫生部“涉及人的生物医学研究伦理审查办法(试行)”(2007 年)，国家中医药管理局“中医药临床研究伦理审查管理规范”(2010 年)，以及世界医学会《赫尔辛基宣言》(2008）等，经本伦理委员会审查，同意按所批准的临床研究方案、知情同意书开展本项研究。		
伦理委员会声明	如发生严重不良事件，应及时报告伦理委员会。<br>如临床试验方案、知情同意书的任何修改，主要研究者更换，应及时通知伦理委员会，重新审查，获得批准后执行。<br>请按照预计预计跟踪审查日期，提交研究进展报告。<br>完成临床研究，请提交结题报告。		
有效期	24 个月	预计跟踪审查日期	2013 年 9 月 20 日
伦理委员会联系人及联系电话		电话：0551-62838532	
伦理委员会主任委员签字及日期		王 2012.9.18	
安徽中医学院第一附属医院（安徽省中医院）伦理委员会（盖章）			
