# Supplementary material for: Factors related to acupuncture response in patients with chronic severe functional constipation: Secondary analysis of a randomized controlled trial
Source: PLoS One. 2017 Nov 22;12(11):e0187723. doi: 10.1371/journal.pone.0187723 (PMC5699843; doi:10.1371/journal.pone.0187723)
Supplement: S1 Table — (DOC) [file pone.0187723.s003.doc]

**Supporting information**

S1 Table. CSBM responder rate from week 1 to week 20

| Week | EA Group | | SA Group | | P Value |
| --- | --- | --- | --- | --- | --- |
|  | n | % | n | % |  |
| Week 1 | 208 | 39.5 | 166 | 31.7 | 0.008 |
| Week 2 | 272 | 51.9 | 184 | 35.5 | <.001 |
| Week 3  Week 4  Week 5  Week 6  Week 7  Week 8  Week 9  Week 10  Week 11  Week 12  Week 13  Week 14  Week 15  Week 16  Week 17  Week 18  Week 19  Week 20 | 308  321  337  340  358  361  373  363  361  354  355  351  337  339  332  339  323  324 | 59.0  61.7  64.9  65.5  69.1  69.7  72.0  70.1  69.7  68.3  68.8  68.0  65.3  65.7  64.3  65.8  62.7  62.9 | 187  201  218  210  232  226  201  218  195  200  202  207  201  197  188  193  192  192 | 36.5  39.3  42.7  41.4  45.8  44.6  39.7  43.1  38.5  39.5  39.9  40.9  39.7  38.9  37.2  38.1  37.9  37.9 | <.001  <.001  <.001  <.001  <.001  <.001  <.001  <.001  <.001  <.001  <.001  <.001  <.001  <.001  <.001  <.001  <.001  <.001 |
